# Consistent Model Selection Procedure for Random Coefficient INAR Models

**DOI:** 10.3390/e25081220

**Published:** 2023-08-16

**Authors:** Kaizhi Yu, Tielai Tao

**Affiliations:** School of Statistics, Southwestern University of Finance and Economics, Chengdu 611130, China; yukz@swufe.edu.cn

**Keywords:** integer-valued time series, model selection, thinning operator, conditional least squares, information criteria

## Abstract

In the realm of time series data analysis, information criteria constructed on the basis of likelihood functions serve as crucial instruments for determining the appropriate lag order. However, the intricate structure of random coefficient integer-valued time series models, which are founded on thinning operators, complicates the establishment of likelihood functions. Consequently, employing information criteria such as AIC and BIC for model selection becomes problematic. This study introduces an innovative methodology that formulates a penalized criterion by utilizing the estimation equation within conditional least squares estimation, effectively addressing the aforementioned challenge. Initially, the asymptotic properties of the penalized criterion are derived, followed by a numerical simulation study and a comparative analysis. The findings from both theoretical examinations and simulation investigations reveal that this novel approach consistently selects variables under relatively relaxed conditions. Lastly, the applications of this method to infectious disease data and seismic frequency data produce satisfactory outcomes.

## 1. Introduction

Integer-valued time series are ubiquitous in scientific research and everyday life, encompassing examples such as the daily count of hospitalized patients admitted to hospitals and the frequency of crimes committed daily or monthly. Consequently, integer-valued time series have increasingly garnered attention from scholars. However, traditional continuous-valued time series models fail to capture the integer-valued characteristics, only approximating integer-valued data through continuous-valued time series models. This approximation may result in model misspecification issues, complicating statistical inference. As a result, the modeling and analysis of integer-valued time series data have become a growing area of focus in academia. Among the variety of integer-valued time series modeling methods, thinning operator models have gained favor due to their resemblance to autoregressive moving average (ARMA) models found in traditional continuous-valued time series theory. Thinning operator models substitute the multiplication in ARMA models with the binomial thinning operator introduced by Steutel and Van Harn [1]:

(1) $$\phi \circ Y_{i}=\sum_{i=1}^{Y_{i}}B_{i}$$

In this equation, $Y_{i}$ represents a count sequence, while $\left\{B_{i}\right\}$ denotes a series of Bernoulli random variables independent of $\left\{Y_{i}\right\}$. The probability mass function satisfies $P\left(B_{i}=1\right)=1-P\left(B_{i}=0\right)=\phi$ with ϕ∈[0,1). Building on this foundation, Al-Osh and Alzaid [2] developed the first-order integer-valued autoregressive (INAR (1)) model for $t\in N^{+}$:

(2) $$Y_{t}=\phi \circ Y_{t-1}+Z_{t}$$

where $Z_{t}$ is regarded as the innovation term entering the model at period t, with its marginal distribution being a Poisson distribution with an expected value of λ. Consequently, model (2) is called the Poisson INAR(1) model. Later, Du and Li [3] introduced the INAR(p) model and provided conditions for ensuring the stationarity and ergodicity of the INAR(p) process. The incorporation of additional lag terms increased the model’s flexibility. Subsequently, Joe [4] and Zheng, Basawa, and Datta [5] developed the random coefficient thinning operator model (RCINAR(1)) by allowing the parameter ϕ in the INAR(1) model to follow a specific random distribution. Zheng, Basawa, and Datta [6] extended the RCINAR(1) model to the p-th order integer-valued autoregressive model, known as the RCINAR(p) model. Zhang, Wang, and Zhu [7] established a data-driven empirical likelihood interval estimation for the RCINAR(p) model using the empirical likelihood (EL) estimation method. By employing the geometric thinning operator (also referred to as the negative binomial thinning operator) proposed by Ristić, Bakouch, and Nastić [8], Tian, Wang, and Cui [9]) constructed an INAR(1) model capable of describing seasonal effects. Lu [10] investigated the prediction problem of the thinning operator model using the Taylor expansion. For further discussions on thinning operator models, readers can consult the textbook by Weiß [11].

In general, researchers engaged in statistical analysis, particularly during the initial stages of time series data investigation, frequently encounter the challenge of model selection. Current model selection techniques can be broadly categorized into three groups: The first group relies on sample autocorrelation (ACF) and partial autocorrelation (PACF) functions for model selection, as exemplified by Latour [12]; the second group, which is the most prevalent method for variable selection, comprises a series of information criteria founded on maximum likelihood estimation. Akaike [13] introduced the Akaike Information Criterion (AIC) by performing an unbiased estimation of the expected log-likelihood function, while Schwarz [14] established the Bayesian Information Criterion (BIC) by employing a Laplace expansion for the posterior estimation of the expected log-likelihood function. Ding, Tarokh, and Yang [15] devised a novel information criterion for autoregressive time series models by connecting AIC and BIC. Furthermore, given that empirical likelihood estimation can substantially circumvent issues stemming from model misspecification and maintain certain maximum likelihood estimation features, researchers have started to investigate data-driven information criteria based on empirical likelihood estimation. Variyath, Chen, and Abraham [16] formulated the Empirical Akaike Information Criterion (EAIC) and the Empirical Bayesian Information Criterion (EBIC) by drawing on the principles of AIC and BIC with empirical likelihood estimation. They demonstrated that EBIC possesses consistency in variable selection. Chen, Wang, Wu, and Li [17] addressed potential computational convergence problems in empirical likelihood estimation by incorporating external estimators (typically moment estimators) into the empirical likelihood function, thereby developing a robust and consistent information criterion. For additional discussions on information criteria, readers may consult the textbook by Konishi and Kitagawa [18] and the review article by Ding, Tarokh, and Hong [19].

In the specific domain of integer-valued time series analysis, our objective is to determine which lagged variables of $Y_{t}$ ought to be incorporated into the model. Extensive research has been conducted on model selection for integer-valued autoregressive conditional heteroskedasticity (INARCH) models, which allow for relatively straightforward likelihood function establishment. Notable examples include Weiß and Feld [20], who provided comprehensive numerical simulations for integer-valued time series model selection using information criteria, and Diop and Kengne [21], who introduced consistent model selection methods for INARCH models based on quasi-maximum likelihood estimation. However, the process becomes more challenging when dealing with higher-order and random coefficient INAR(p) models constructed using thinning operators. The complexity of the likelihood functions and the substantial computational requirements make it difficult to establish and utilize information criteria. Consequently, Zheng, Basawa, and Datta [6] proposed estimating the model based on its conditional moments rather than relying on likelihood functions. While this approach facilitates the estimation of unknown parameters for researchers, it creates complications for variable selection. To overcome this hurdle, Wang, Wang, and Yang [22] implemented penalty functions and pseudo-quasi-maximum likelihood estimation (PQML) for variable selection, demonstrating the robustness of their method even when faced with contaminated data. Drawing inspiration from these preceding studies, this paper endeavors to establish a novel model selection method akin to information criteria founded upon the estimating equations in conditional least squares (CLS) estimation. Furthermore, we attempt to demonstrate the consistency of this innovative model selection method in addressing variable selection problems within integer-valued time series. This approach circumvents the need for complex probability distribution assumptions while preserving effective variable selection capabilities.

The organization of this paper is as follows: In Section 2, we revisit the RCINAR(p) model, introduce the proposed information criterion, and outline its asymptotic properties. In Section 3, we carry out numerical simulation studies on variable selection utilizing this information criterion. In Section 4, we endeavor to apply this information criterion for variable selection in real data sets. Lastly, in Section 5, we engage in a discussion and offer concluding remarks.

## 2. RCINAR Model and Model Selection Procedure

In this section, we discuss the ergodic stationary RCINAR model and its associated model selection methods.

### 2.1. RCINAR(p) Model and Its Estimation

The INAR(p) model with constant coefficients, as introduced by Du and Li [3], is formulated as follows:

(3) $$Y_{t}=\phi_{1}\circ Y_{t-1}+\cdots +\phi_{p}\circ Y_{t-p}+Z_{t}$$

In this expression, given the vector ${\left(Y_{t-1},\cdots ,Y_{t-p}\right)}^{\prime }$, the elements ${\left(\phi_{1}\circ Y_{t-1},\cdots ,\phi_{p}\circ Y_{t-p}\right)}^{\prime }$ are deemed to be mutually conditionally independent. This conditional independence ensures that the autocorrelation function of the INAR(p) model is congruent with that of its continuous-valued Autoregressive (AR(p)) counterpart. Moreover, Du and Li [3] substantiated that, under these model settings, the stationarity condition for the INAR(p) model necessitates that the roots of the polynomial $h\left(z\right)=1-\phi_{1}z-\cdots -\phi_{p}z^{p}=0$ are located outside the unit circle. This implies that the INAR(p) model attains stationarity when the sum $\sum_{i=1}^{p}\phi_{i}$ is less than 1. Building upon these foundational insights, Zheng, Basawa, and Datta [6] extended the INAR(p) model under the constant coefficient assumption, giving rise to the Random Coefficient Integer-valued Autoregressive (RCINAR(p)) model.

Let ${\left\{Y_{t}\right\}}_{t=1}^{T}$ represent a non-negative integer-valued sequence. The RCINAR(p) model is defined by the following equation:

(4) $$Y_{t}=\phi_{1}^{\left(t\right)}\circ Y_{t-1}+\cdots +\phi_{p}^{\left(t\right)}\circ Y_{t-p}+Z_{t}$$

where “∘” denotes the thinning operator defined in Equation (1). Let $\theta_{0}={\left(\phi_{10},\ldots ,\phi_{p0},\lambda_{0}\right)}^{\prime }$ be the true parameter vector of this data-generating process, with $\theta_{0}\in \Theta$, where Θ is a compact subset of $R^{p+1}$. $\theta ={\left(\phi_{1},\ldots ,\phi_{p},\lambda \right)}^{\prime }$ represents the p+1 dimensional parameter vector to be estimated. Here, $\left\{\phi_{j}^{\left(t\right)}\right\}$ are sequences of independent and identically distributed random variables defined on [0,1) with a mean of $\phi_{j}$, and their probability density function $f_{\phi_{j}}\left(\phi \right)\geq 0$, ∀ϕ∈[0,1), with $\sum_{j=1}^{p}\phi_{j}<1$. 

Moreover, we do not assume a specific parametric distribution for $\left\{Z_{t}\right\}$, only requiring that $\left\{Z_{t}\right\}$ be an independent and identically distributed non-negative integer-valued random variable sequence with a mean of λ and a probability mass function $f_{Z}\left(z\right)\geq 0$, ∀z∈N. In this context, we consider the semiparametric INAR model as described by Drost, Van den Akker, and Werker [23].

**Remark** **1.** *As can be discerned from the preceding discussion, the INAR(p) model (3) represents a special case of the RCINAR(p) model (4). That is, when  *$\left\{\phi_{j}^{\left(t\right)}\right\}$ *is a constant coefficient vector, the RCINAR(p) model reduces to the INAR(p) model. As demonstrated by Zheng, Basawa, and Datta [6], the statistical methods employed in the study of the RCINAR(p) model can also be directly applied to the INAR(p) model. Consequently, in order to cater to a wider range of application scenarios, the academic community tends to prioritize the study of the RCINAR model while investigating thinning operator models. For instance, Kang and Lee [24] investigated the problem of change-point detection in the RCINAR model by leveraging the Cumulative Sum (CUSUM) test. Similarly, Zhang, Wang, and Zhu [7] proposed an interval estimation method for the RCINAR model based on empirical likelihood estimation. Awale, Balakrishna, and Ramanathan [25], on the other hand, constructed a locally most powerful-type test devised specifically for examining structural changes within the framework of the RCINAR model. Therefore, this paper will center its research on the RCINAR model.*

To estimate the RCINAR(p) model and establish model selection criteria, we draw inspiration from the assumptions delineated by Zhang, Wang, and Zhu [7]. These assumptions are as follows:

(A1) $\left\{Y_{t}\right\}$ constitutes an ergodic and strictly stationary RCINAR(p) process.
(A2) There exists δ>0 such that $E{\left|Y_{t}\right|}^{4+\delta }<\infty$.

Derived from Equation (4), the one-step-ahead transition probability is as follows:

PYt=iYt−1=i1,⋯,Yt−p=ip=∑k=0min⁡i,∑j=1pijfZi−k∏0≤∑j=1pkj≤kijkj×∫0≤ϕ1t≤⋯≤ϕpt<1∏j=1pϕjtkj1−ϕjtij−kjdPϕ1t,⋯,ϕpt

Here, $P_{\phi_{1}^{\left(t\right)},\cdots ,\phi_{p}^{\left(t\right)}}$ represents the joint distribution function of $\phi_{1}^{\left(t\right)},\cdots ,\phi_{p}^{\left(t\right)}$. Utilizing this one-step-ahead transition probability function, we can construct the likelihood function:

$$L=P\left(Y_{p}=i_{1}^{\left(p+1\right)},\cdots ,Y_{1}=i_{p}^{\left(p+1\right)}\right)\prod_{t=p+1}^{T}P\left(Y_{t}=i^{\left(t\right)}|Y_{t-1}=i_{1}^{\left(t\right)},\cdots ,Y_{t-p}=i_{p}^{\left(t\right)}\right)$$

The likelihood function L for model (4) is notably complex, involving numerous multivariate numerical integrations within statistical computations, which demand substantial computational resources. Consequently, Zheng, Basawa, and Datta [6] advocated for estimating the model based on its conditional moments rather than employing the likelihood function. This preference also underlies the prevalent use of conditional least squares (CLS) estimation in the study of RCINAR(p) models within the scholarly community. In the subsequent section, we offer a concise introduction to the CLS estimation methodology for the RCINAR(p) model.

We can obtain the first-order conditional moment of model (4) as follows:

$$E\left(Y_{t}|F_{t-1}\right)=\sum_{j=1}^{p}\phi_{j}Y_{t-j}+\lambda$$

where $F_{t-1}=\sigma (Y_{t-1},Y_{t-2},\cdots )$. This derivation allows us to compute the conditional least squares (CLS) estimation. Let

$$S\left(\theta \right)=\sum_{t=p+1}^{T}{\left(Y_{t}-\sum_{j=1}^{p}\phi_{j}Y_{t-j}-\lambda \right)}^{2}$$

represent the conditional least squares (CLS) objective function. The CLS estimator is then given by:

$$\hat{\theta }=argmin_{\theta }\left(S\left(\theta \right)\right)$$

Let

$$S_{t}\left(\theta \right)={\left(Y_{t}-\sum_{j=1}^{p}\phi_{j}Y_{t-j}-\lambda \right)}^{2}$$

Then the estimating equations are:

$$-\frac{1}{2}\frac{\partial S_{t}\left(\theta \right)}{\partial \theta }=0=\Psi_{t}\left(\theta \right)={\left(\psi_{t}^{\left(1\right)}\left(\theta \right),\psi_{t}^{\left(2\right)}\left(\theta \right),\ldots ,\psi_{t}^{\left(p+1\right)}\left(\theta \right)\right)}^{\prime }$$

where

$$\psi_{t}^{\left(s\right)}=\left(Y_{t}-\sum_{j=1}^{p}\phi_{j}Y_{t-j}-\lambda \right)Y_{t-s},\;1\leq s\leq p$$

$$\psi_{t}^{\left(p+1\right)}=Y_{t}-\sum_{j=1}^{p}\phi_{j}Y_{t-j}-\lambda$$

For the estimating equation $\Psi_{t}\left(\theta \right)$, we introduce an additional assumption:

(A3) $\Psi_{t}\left(\theta \right)$ is identifiable, that is, $E\left(\Psi_{t}\left(\theta_{0}\right)\right)=0$, and if θ is in the neighborhood of $\theta^{*}\neq \theta_{0}$, then $\left\|E\left(\Psi_{t}\left(\theta \right)\right)\right\|$ exists and $\left\|E\left(\Psi_{t}\left(\theta \right)\right)\right\|>0$.

Assumption (A3) is the identifiability assumption, which further implies that the model (4) is identifiable if only the currently specified model satisfies $E\left(\Psi_{t}\left(\theta_{0}\right)\right)=0$. Based on these assumptions above, the following lemma can be deduced:

**Lemma** **1.** *Based on assumptions (A1) to (A3), the subsequent conclusions are valid:*

(i) $E(\Psi_{t}\left(\theta_{0}\right)\Psi_{t}{\left(\theta_{0}\right)}^{\prime })$ *constitutes a positive definite matrix.*
(ii) $\frac{\partial^{2}\Psi_{t}\left(\theta \right)}{\partial \theta \partial \theta^{\prime }}$ *remains continuous within the neighborhood of* $\theta_{0}$.
(iii) *Both*  $\left\|\frac{\partial \Psi_{t}\left(\theta \right)}{\partial \theta^{\prime }}\right\|$ *and*  $\left\|\frac{\partial^{2}\Psi_{t}\left(\theta \right)}{\partial \theta \partial \theta^{\prime }}\right\|$ *possess upper bounds in the neighborhood of* $\theta_{0}$.

Moreover, Zheng, Basawa, and Datta [6] established that ${\hat{\theta }}_{CLS}$ is a consistent estimator with an asymptotic distribution:

(5) $$\sqrt{T}\left(\hat{\theta }-\theta \right)\overset{d}{\to }N(V^{-1}(\theta_{0})W(\theta_{0})V^{-1}(\theta_{0}))$$

where:

$$W\left(\theta_{0}\right)=E\left(\Psi_{t}\left(\theta_{0}\right)\Psi_{t}{\left(\theta_{0}\right)}^{\prime }\right)$$

$$V\left(\theta_{0}\right)=E\left(\frac{\partial E\left(Y_{t}|Y_{t-1}\right)}{\partial \theta }\cdot \frac{\partial E\left(Y_{t}|Y_{t-1}\right)}{\partial \theta^{\prime }}\right)-E\left(u_{t}\left(\theta_{0}\right)\frac{\partial^{2}E\left(Y_{t}|Y_{t-1}\right)}{\partial \theta \partial \theta^{\prime }}\right)$$

$$u_{t}\left(\theta_{0}\right)=Y_{t}-E\left(Y_{t}|Y_{\left(t-1\right)}\right)$$

### 2.2. Model Selection Procedure

For the data-generating process defined by Equation (4), we establish the following settings:

1. A model m is a subset of M={1,2,…,p,p+1}, with its dimension denoted as |m|. Consequently, p+1 represents the maximum model dimension we consider, noted as the full model, while the minimum model dimension we consider is 1, corresponding to an independent and identically distributed non-negative integer-valued random variable sequence. Let the true model be $m_{0}$.
2. $\theta \left(m\right)$ is the parameter vector associated with model m, which can be extended to the p + 1 dimensional vector $\tilde{\theta }\left(m\right)=\left\{{\left(\theta_{j}\right)}_{1\leq j\leq p+1}:\theta_{j}=\theta {\left(m\right)}_{j},if\;j\in m;\theta_{j}=0,if\;j\notin m\right\}$. For instance, if the considered model m is $Y_{t}=\phi_{1}^{\left(t\right)}\circ Y_{t-1}+\phi_{3}^{\left(t\right)}\circ Y_{t-3}+Z_{t}$, then m={1,3,p+1}, $\theta \left(m\right)=(\phi_{1},\phi_{3},\lambda )$, and it can be extended to the p+1 dimensional vector $\tilde{\theta }\left(m\right)=(\phi_{1},0,\phi_{3},0,\ldots ,0,\lambda )$.
3. Let Θ(m) be the compact parameter space of model m, $\tilde{\Theta }\left(m\right)=\left\{{\left(\theta_{j}\right)}_{1\leq j\leq p+1}\in R^{p+1}:\theta_{j}=0,if\;j\notin m\right\}$ constitutes a compact subset of $R^{p+1}$, and all possible $\tilde{\theta }(m)$ values, when restricted to the |m| dimensional vector $\theta \left(m\right)$, are interior points of its corresponding compact subset Θ(m). Furthermore, we denote $\tilde{\theta }=\theta \left(M\right)$ as the parameter vector to be estimated in $\tilde{\Theta }\left(M\right)=\Theta (M)$, i.e., the parameter vector of the full model M.

For model m, we partition $\tilde{\theta }$ into two components, i.e., $\tilde{\theta }={\left({\tilde{\theta }}_{\left(1\right)}^{}{\left(m\right)}^{\prime },{\tilde{\theta }}_{\left(2\right)}^{}{\left(m\right)}^{\prime }\right)}^{\prime }$, where ${\tilde{\theta }}_{\left(1\right)}\left(m\right)=\left\{\left(\theta_{j}\right),j\in m\right\}=\theta \left(m\right)$ and ${\tilde{\theta }}_{\left(2\right)}\left(m\right)=\left\{\left(\theta_{j}\right),j\notin m\right\}$. Correspondingly, it is evident that if the model m is correctly specified, denoted as $m=m_{0}$, ${\tilde{\theta }}_{\left(2\right)}^{}\left(m_{0}\right)=0$, then ${\tilde{\theta }}_{0}={\left({\tilde{\theta }}_{\left(1\right)}^{}{\left(m_{0}\right)}^{\prime },{\tilde{\theta }}_{\left(2\right)}^{}{\left(m_{0}\right)}^{\prime }\right)}^{\prime }={\left(\theta {\left(m_{0}\right)}^{\prime },0^{\prime }\right)}^{\prime }$. We can then divide the estimating equation $\Psi_{t}\left(\tilde{\theta }\right)$ into two parts:

$$\Psi_{t}\left(\tilde{\theta }\right)=\left\{\begin{array}{c} \Psi_{1t}\left(\tilde{\theta }\right)\\ \Psi_{2t}\left(\tilde{\theta }\right) \end{array}\right.$$

where

$$\Psi_{1t}\left(\tilde{\theta }\right)=-\frac{1}{2}\frac{\partial S_{t}\left(\tilde{\theta }\right)}{\partial {\tilde{\theta }}_{\left(1\right)}(m)}$$

$$\Psi_{2t}\left(\tilde{\theta }\right)=-\frac{1}{2}\frac{\partial S_{t}\left(\tilde{\theta }\right)}{\partial {\tilde{\theta }}_{\left(2\right)}(m)}$$

Let ${\hat{\theta }}_{CLS}\left(m\right)={\left({\hat{\theta }}_{\left(1\right),CLS}^{\prime }\left(m\right),0^{\prime }\right)}^{\prime }$, i.e., ${\hat{\theta }}_{\left(1\right),CLS}^{}\left(m\right)$ is the solution to $\Psi_{1t}\left({\tilde{\theta }}_{\left(1\right)}\left(m\right),0\right)=0$, where ${\tilde{\theta }}_{\left(2\right)}^{}\left(m\right)$ is constrained to be 0. Therefore ${\hat{\theta }}_{\left(1\right),CLS}^{}\left(m\right)$ represents the CLS estimator of model m. Define the function:

$$H\left(\tilde{\theta }\right)={\left(\sum_{t=p+1}^{T}\Psi_{t}\left(\tilde{\theta }\right)\right)}^{\prime }{\left(\sum_{t=p+1}^{T}\Psi_{t}\left(\tilde{\theta }\right)\Psi_{t}{\left(\tilde{\theta }\right)}^{\prime }\right)}^{-1}\left(\sum_{t=p+1}^{T}\Psi_{t}\left(\tilde{\theta }\right)\right)$$

We can then derive the following lemma:

**Lemma** **2.** *Given assumptions (A1)–(A3), as* T→∞*:*

$$H\left({\tilde{\theta }}_{0}\right)\to \chi_{p+1}^{2}$$

Because the proof of this lemma closely resembles the proof of Theorem 1 in Zhang, Wang, and Zhu [7], we omit the details. It is important to note that when m=M, ${\hat{\theta }}_{CLS}\left(M\right)$ is the solution to the estimating equation $\sum_{t=p+1}^{T}\Psi_{t}\left(\tilde{\theta }\right)=0$, and in this case, $H\left({\hat{\theta }}_{CLS}\left(M\right)\right)=0$. Furthermore, Lemma 2 suggests that $H\left({\tilde{\theta }}_{0}\right)=O_{p}(1)$.

**Definition** **1.** *We propose the following penalized criteria:*

(6) $$H\left({\hat{\theta }}_{CLS}\left(m\right)\right)+P_{T}\cdot \left|m\right|$$

*where the penalty term* $P_{T}$* is an increasing sequence*, $P_{T}\to \infty$* and satisfies* $P_{T}=O\left(T^{\frac{1}{2}}\right)$ and $\frac{log(T)}{P_{T}}=O\left(1\right)$.

**Remark** **2.** *Intuitively, in this penalized criterion,* $H\left({\hat{\theta }}_{CLS}\left(m\right)\right)$ *serves as a measure of the model’s fit to the data. If it can be demonstrated that the divergence rate of* $H\left({\hat{\theta }}_{CLS}\left(m\right)\right)$ *is slower when* $m_{0}\subseteq m$ *compared to the divergence rate of* $H\left({\hat{\theta }}_{CLS}\left(m_{1}\right)\right)$ *when* $m_{0}⊈m_{1}$*, then a smaller* $H\left({\hat{\theta }}_{CLS}\left(m\right)\right)$ *would suggest a superior fit of model* m  *to the data. However, upon closer examination, it becomes evident that if we merely adopt model* M*, then* $H\left({\hat{\theta }}_{CLS}\left(M\right)\right)=0$ *. Consequently, it is necessary to introduce a penalty term,* $P_{T}\cdot \left|m\right|$*, to constrain the number of lagged variables incorporated by model* m*. By striking a balance between the degree of data fitting* $H\left({\hat{\theta }}_{CLS}\left(m\right)\right)$ *and the number of lagged variables* $P_{T}\cdot \left|m\right|$*, Theorems 1–3 substantiate the ability to select the appropriate model.*

Under the correct model specification, the following theorem can be derived:

**Theorem** **1.** *Given assumptions (A1) and (A2), under the correct model specification*

$${\hat{\theta }}_{\left(1\right),CLS}^{}\left(m\right)-{\tilde{\theta }}_{\left(1\right)}\left(m_{0}\right){=-\left(E\left(\frac{\partial \Psi_{t}\left({\tilde{\theta }}_{0}\right)}{\partial {\tilde{\theta }}_{\left(1\right)}^{\prime }\left(m\right)}\right)\right)}^{-1}\frac{1}{T}\sum_{t=p+1}^{T}\Psi_{1t}\left({\tilde{\theta }}_{0}\right)+o_{p}\left(T^{-\frac{1}{2}}\right)$$

*and* $H\left({\hat{\theta }}_{CLS}\left(m\right)\right)$ *converges in probability to* $\sum_{j=1}^{p+1}\Lambda_{j}\chi^{2}(1)$, where $\Lambda_{j}$ *is the eigenvalue of the matrix* $\Sigma_{11}^{\frac{1}{2}}\Sigma_{*}^{\prime }\Sigma_{11}^{-1}\Sigma_{*}\Sigma_{11}^{\frac{1}{2}}$, *where*

$$\Sigma_{*}=\left(I-E\left(\frac{\partial \Psi_{t}\left({\tilde{\theta }}_{0}\right)}{\partial {\tilde{\theta }}^{\prime }}\right)\left[\begin{array}{cc} {\left(E\left(\frac{\partial \Psi_{t}\left({\tilde{\theta }}_{0}\right)}{\partial {\tilde{\theta }}_{\left(1\right)}^{\prime }\left(m\right)}\right)\right)}^{-1} & 0\\ 0 & 0 \end{array}\right]\right)$$

$$\Sigma_{11}=E\left(\Psi_{t}\left({\tilde{\theta }}_{0}\right)\Psi_{t}{\left({\tilde{\theta }}_{0}\right)}^{\prime }\right)$$

Theorem 1 establishes the asymptotic distribution of ${\hat{\theta }}_{\left(1\right),CLS}^{}\left(m\right)$ and $H\left({\hat{\theta }}_{CLS}\left(m\right)\right)$ under the correct model specification, which serves as a crucial component in the derivation for the consistency of our penalized criteria (6). In the following, we discuss the performance of $H\left({\hat{\theta }}_{CLS}\left(m\right)\right)$ when the model specification m is incorrect.

**Theorem** **2.** *Given assumptions (A1)–(A3), for any* ${\tilde{\theta }}_{1}$ *in the neighborhood of* ${\tilde{\theta }}^{*}\neq {\tilde{\theta }}_{0}$*, we have:*

$$T^{-\frac{1}{2}}H\left({\tilde{\theta }}_{1}\right)\to \infty$$

Theorem 2 and assumption (A3) ensure that if the model m is misspecified, $H\left({\hat{\theta }}_{CLS}\left(m\right)\right)$ will diverge to positive infinity at a rate of at least $T^{\frac{1}{2}}$. Combining Theorems 1 and 2, we can present the primary conclusion of this paper. When the model is specified as m, we have the following theorem.

**Theorem** **3.** *Given assumptions (A1)–(A3), we have:*

$$P\left(\min\left(H\left({\hat{\theta }}_{CLS}\left(m\right)\right)+P_{T}\cdot \left|m\right|:m\neq m_{o}\right)>H\left({\hat{\theta }}_{CLS}\left(m_{0}\right)\right)+P_{T}\cdot \left|m_{0}\right|\right)\to 1$$

From the proof of Theorem 3, and Lemma A.1, we can observe that the divergence rate of $P_{T}$ needs to be at least as fast as log⁡(T). In practical applications, we may use settings such as $P_{T}=T^{\frac{1}{5}}$. In such settings, although $\frac{log(T)}{P_{T}}\to 0$, in finite samples, $P_{T}<log(T)$. In fact, in the interval [4,332,106], $T^{\frac{1}{5}}<log(T)$, which may result in the performance of $P_{T}=T^{\frac{1}{5}}$ not being as effective as $P_{T}=log(T)$ in finite samples. Nevertheless, such penalty term settings still hold value, and we will discuss this situation in the numerical simulation section.

Theorem 3 provides the consistency of the penalized criteria (6) for model selection. It becomes evident that Theorem 3 holds under very relaxed assumptions and relies solely on the CLS estimation, which can be rapidly completed in any statistical software, and the estimating equation constructed by first-order conditional moments, which is easy to derive. This makes the penalized criteria (6) highly suitable for use in INAR models, particularly in RCINAR models. Now let $\hat{m}$ be the model selected by the criterion (6):

$$\hat{m}=argmin_{m\subseteq M}\left(H\left(m\right)+P_{T}\cdot \left|m\right|\right)$$

We now present the asymptotic properties of the selected model:

**Theorem** **4.** *Given assumptions (A1)–(A3), we have:*

$$\sqrt{T}({\hat{\theta }}_{CLS}\left(\hat{m}\right)-{\tilde{\theta }}_{0})\overset{d}{\to }N(V^{-1}({\tilde{\theta }}_{0})W({\tilde{\theta }}_{0})V^{-1}({\tilde{\theta }}_{0}))$$

*where:*

$$W\left({\tilde{\theta }}_{0}\right)=E\left(\Psi_{t}\left({\tilde{\theta }}_{0}\right)\Psi_{t}{\left({\tilde{\theta }}_{0}\right)}^{\prime }\right)$$

$$V\left({\tilde{\theta }}_{0}\right)=E\left(\frac{\partial E\left(Y_{t}|Y_{t-1}\right)}{\partial \tilde{\theta }}\cdot \frac{\partial E\left(Y_{t}|Y_{t-1}\right)}{\partial {\tilde{\theta }}^{\prime }}\right)-E\left(u_{t}\left({\tilde{\theta }}_{0}\right)\frac{\partial^{2}E\left(Y_{t}|Y_{t-1}\right)}{\partial \tilde{\theta }\partial {\tilde{\theta }}^{\prime }}\right)$$

$$u_{t}\left({\tilde{\theta }}_{0}\right)=Y_{t}-E\left(Y_{t}|Y_{\left(t-1\right)}\right)$$

**Remark** **3.** *From the inference process in this section, we can see that the estimating equation used in constructing the penalized criteria (6) actually utilizes the information of* $E(Y_{t}|F_{t-1})$, where $F_{t-1}=\sigma (Y_{t-1},Y_{t-2},\cdots )$ *and does not involve the information of thinning operators. Therefore, the penalized criteria (6) can be applied to models with the same linear form conditional expectations, such as INARCH models and continuous-valued AR models. The likelihood functions of INARCH and AR models can be established with relative ease, enabling us to compare the efficacy of the penalty criteria (6) with that of AIC and BIC across both models.*

## 3. Numerical Simulations

In this section, we first conduct a simulation study to evaluate the performance of the penalized criteria proposed in this paper for INAR models. Secondly, to compare the proposed penalized criteria with the traditional likelihood-based AIC and BIC, we apply these criteria to INARCH models and AR models. Finally, by utilizing innovation terms of different random distributions, we carry out a simulation study on the robustness of the penalized criteria proposed in this paper.

### 3.1. Performance of the Penalized Criteria in INAR Models

In this subsection, we consider the true data-generating process to be:

(7) $$Y_{t}=\phi_{1}^{\left(t\right)}\circ Y_{t-1}+\phi_{3}^{\left(t\right)}\circ Y_{t-3}+Z_{t}$$

where the mean of $\phi_{1}^{\left(t\right)}$ is 0.4, the mean of $\phi_{3}^{\left(t\right)}$ is 0.2, and λ=2, i.e., ${\tilde{\theta }}_{0}={\left(0.4,0,0.2,2\right)}^{\prime }$. By applying the penalized criteria (6), we attempt to select the true model from all RCINAR models up to the third order. In Table 1 below:

i.i.d. represents an *i.i.d. Poisson* random variable sequence,
$y_{t-1}$ represents the model $Y_{t}=\phi_{1}^{\left(t\right)}\circ Y_{t-1}+Z_{t}$,
$y_{t-2}\;represents\;the\;model\;Y_{t}=\phi_{2}^{\left(t\right)}\circ Y_{t-2}+Z_{t}$,
$y_{t-3}\;represents\;the\;model\;Y_{t}=\phi_{3}^{\left(t\right)}\circ Y_{t-3}+Z_{t}$,
$y_{t-1}{,y}_{t-2}\;represents\;the\;model\;Y_{t}=\phi_{1}^{\left(t\right)}\circ Y_{t-1}+\phi_{2}^{\left(t\right)}\circ Y_{t-2}+Z_{t}$,
$y_{t-1}{,y}_{t-3}\;represents\;the\;model\;Y_{t}=\phi_{1}^{\left(t\right)}\circ Y_{t-1}+\phi_{3}^{\left(t\right)}\circ Y_{t-3}+Z_{t}$,
$y_{t-1}{,y}_{t-3}\;represents\;the\;model\;Y_{t}=\phi_{2}^{\left(t\right)}\circ Y_{t-2}+\phi_{3}^{\left(t\right)}\circ Y_{t-3}+Z_{t}$,
$y_{t-1}{,y}_{t-2}{,y}_{t-3}\;represents\;the\;model\;Y_{t}=\phi_{1}^{\left(t\right)}\circ Y_{t-1}+\phi_{2}^{\left(t\right)}\circ Y_{t-2}+\phi_{3}^{\left(t\right)}\circ Y_{t-3}+Z_{t}$.

In addition, “Coef” denotes the random distribution of the coefficient. In this subsection, we focus on the performance of penalized criteria in INAR models. We use boldface to highlight the true model, i.e., $y_{t-1},y_{t-3}$. We compare three different penalty term settings $P_{T}=log(T)$, $P_{T}=T^{\frac{1}{3}}$, and $P_{T}=T^{\frac{1}{5}}$ and consider three different distributions for $\phi_{1}^{\left(t\right)}$ and $\phi_{3}^{\left(t\right)}$:

(i) Fixed coefficients, i.e., $\phi_{1}^{\left(t\right)}=0.4$, $\phi_{3}^{\left(t\right)}=0.2$, regardless of t;
(ii) $\phi_{1}^{\left(t\right)}$ follows a uniform distribution on the interval [0,0.8], $\phi_{3}^{\left(t\right)}$ follows a uniform distribution on the interval [0,0.4];
(iii) $\phi_{1}^{\left(t\right)}$ follows a beta distribution with a mean of 0.4, $\phi_{3}^{\left(t\right)}$ follows a beta distribution with a mean of 0.2. In this scenario, we fix the parameter vector (a,b) for the beta distribution with a=4 and control the parameter b to achieve different means.

We consider sample sizes *T* = 100, 200, 300, 500, 1000, and for each sample size T and parameter setting, we perform 1000 independent repeated experiments.

As shown in Table 1, for the three penalty terms, the accuracy of model selection using the penalized criteria (6) increases with the sample size T, consistent with the asymptotic conclusion described in Theorem 3. However, when the sample size is large, we find that the accuracy of $P_{T}=T^{\frac{1}{5}}$ is slightly worse than $P_{T}=T^{\frac{1}{3}}$ and $P_{T}=log(T)$. This is because

$$\frac{T^{1/5}}{log(T)}\to \infty$$

However, in the interval [4,332,106], $T^{\frac{1}{5}}<log(T)$, which may cause the performance of $P_{T}=T^{\frac{1}{5}}$ in larger finite samples to be not as good as $P_{T}=log(T)$. Nonetheless, the penalty term setting $P_{T}=T^{\frac{1}{5}}$ is not entirely without merit. As shown in Table 1, when the sample size is small, i.e., T≤500, the performance of $P_{T}=T^{\frac{1}{5}}$ is better.

**Table 1:** Frequency of model selection for INAR model of order 2 by the penalized criterion (6).

$Y_{t}=\phi_{1}^{\left(t\right)}\circ Y_{t-1}+\phi_{3}^{\left(t\right)}\circ Y_{t-3}+Z_{t}$										
$\phi_{1}=0.4$ $,\;\phi_{3}=0.2$			Models to be Selected							
T	Coef	$P_{T}$	i.i.d.	$y_{t-1}$	$y_{t-2}$	$y_{t-3}$	$y_{t-1}{,y}_{t-2}$	$y_{t-1}{,y}_{t-3}$	$y_{t-2}{,y}_{t-3}$	$y_{t-1}{,y}_{t-2}{,y}_{t-3}$
100	Fixed	$T^{1/3}$	0.054	0.601	0.003	0.019	0.015	**0.296**	0.002	0.01
		log(T)	0.05	0.599	0.003	0.018	0.016	**0.301**	0.002	0.011
		$T^{1/5}$	0.006	0.361	0.001	0.012	0.047	**0.493**	0.001	0.079
	Uniform	$T^{1/3}$	0.059	0.596	0.005	0.037	0.015	**0.275**	0	0.013
		log(T)	0.055	0.592	0.005	0.037	0.015	**0.283**	0	0.013
		$T^{1/5}$	0.01	0.366	0.001	0.018	0.049	**0.475**	0.004	0.077
	Beta	$T^{1/3}$	0.072	0.585	0.002	0.029	0.026	**0.281**	0.002	0.003
		log(T)	0.069	0.582	0.002	0.029	0.027	**0.285**	0.002	0.004
		$T^{1/5}$	0.013	0.369	0.002	0.017	0.056	**0.479**	0.003	0.061
200	Fixed	$T^{1/3}$	0	0.368	0	0.002	0.016	**0.607**	0	0.007
		log(T)	0	0.326	0	0.002	0.017	**0.644**	0	0.011
		$T^{1/5}$	0	0.126	0	0	0.03	**0.781**	0	0.063
	Uniform	$T^{1/3}$	0.001	0.429	0	0.002	0.016	**0.545**	0	0.007
		log(T)	0	0.37	0	0.001	0.02	**0.594**	0	0.015
		$T^{1/5}$	0	0.159	0	0	0.032	**0.721**	0	0.088
	Beta	$T^{1/3}$	0.002	0.363	0	0.001	0.021	**0.602**	0	0.011
		log(T)	0.002	0.314	0	0	0.025	**0.645**	0	0.014
		$T^{1/5}$	0	0.122	0	0	0.029	**0.768**	0	0.081
300	Fixed	$T^{1/3}$	0	0.183	0	0	0.008	**0.802**	0	0.007
		log(T)	0	0.132	0	0	0.007	**0.845**	0	0.016
		$T^{1/5}$	0	0.037	0	0	0.009	**0.88**	0	0.074
	Uniform	$T^{1/3}$	0	0.252	0	0	0.01	**0.725**	0	0.013
		log(T)	0	0.176	0	0	0.015	**0.79**	0	0.019
		$T^{1/5}$	0	0.06	0	0	0.02	**0.842**	0	0.078
	Beta	$T^{1/3}$	0	0.218	0	0	0.012	**0.766**	0	0.004
		log(T)	0	0.15	0	0	0.016	**0.825**	0	0.009
		$T^{1/5}$	0	0.06	0	0	0.021	**0.859**	0	0.06
500	Fixed	$T^{1/3}$	0	0.04	0	0	0.002	**0.955**	0	0.003
		log(T)	0	0.014	0	0	0.003	**0.974**	0	0.009
		$T^{1/5}$	0	0.002	0	0	0.002	**0.95**	0	0.046
	Uniform	$T^{1/3}$	0	0.062	0	0	0.004	**0.932**	0	0.002
		log(T)	0	0.03	0	0	0.007	**0.955**	0	0.008
		$T^{1/5}$	0	0.007	0	0	0.006	**0.919**	0	0.068
	Beta	$T^{1/3}$	0	0.046	0	0	0.003	**0.936**	0	0.015
		log(T)	0	0.026	0	0	0.003	**0.96**	0	0.011
		$T^{1/5}$	0	0.005	0	0	0.003	**0.932**	0	0.06
1000	Fixed	$T^{1/3}$	0	0	0	0	0	**0.999**	0	0.001
		log(T)	0	0	0	0	0	**0.989**	0	0.011
		$T^{1/5}$	0	0	0	0	0	**0.964**	0	0.036
	Uniform	$T^{1/3}$	0	0	0	0	0	**0.997**	0	0.003
		log(T)	0	0	0	0	0	**0.99**	0	0.01
		$T^{1/5}$	0	0	0	0	0	**0.952**	0	0.048
	Beta	$T^{1/3}$	0	0	0	0	0	**0.998**	0	0.002
		log(T)	0	0	0	0	0	**0.992**	0	0.008
		$T^{1/5}$	0	0	0	0	0	**0.94**	0	0.06

**Figure 1:**
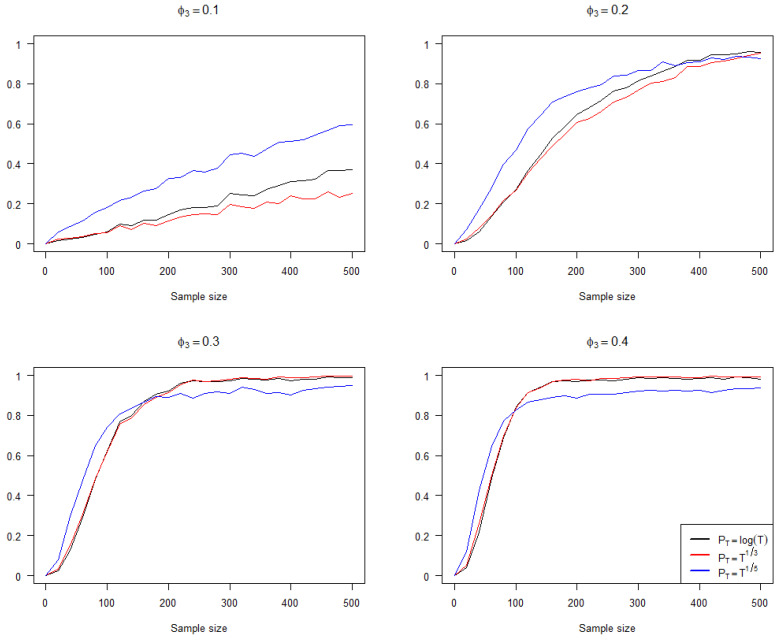
The impact of sample size on accuracy under different $\phi_{3}$ settings.

**Figure 2:**
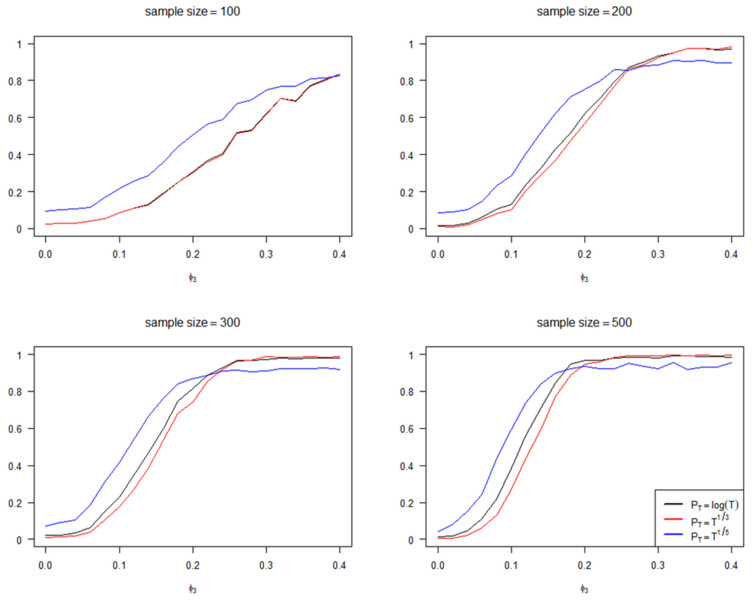
The impact of $\phi_{3}$ settings on accuracy under different sample sizes.

To investigate the performance of the three penalty terms under varying sample sizes and coefficient mean settings, we continue to consider model (7), where $\phi_{1}^{\left(t\right)}$ follows a beta distribution with a mean of 0.4, and $\phi_{3}^{\left(t\right)}$ follows a beta distribution with a mean of $\phi_{3}$. In Figure 1, we report the impact of sample size on the accuracy of the penalized criteria using the three penalty terms under different $\phi_{3}$ settings. In Figure 1 and Figure 2, the red line represents $P_{T}=T^{\frac{1}{3}}$, the black line represents $P_{T}=log(T)$, and the blue line represents $P_{T}=T^{\frac{1}{5}}$, and the vertical axis of both figures represents the frequency of the penalized criteria (6) selecting the correct model. It can be observed that when $\phi_{3}$ is small or the sample size is small, the performance of $P_{T}=T^{\frac{1}{5}}$ is superior. However, as $\phi_{3}$ gradually moves further from 0 and the sample size increases, the performance of $P_{T}=T^{\frac{1}{5}}$ becomes slightly worse than $P_{T}=log(T)$ and $P_{T}=T^{\frac{1}{3}}$.

In Figure 2, we report the frequency of selecting the model $Y_{t}=\phi_{1}^{\left(t\right)}\circ Y_{t-1}+\phi_{3}^{\left(t\right)}\circ Y_{t-3}+Z_{t}$ using the penalized criteria (6) as $\phi_{3}$ gradually varies from 0 to 0.4 under different sample size conditions. It should be noted that when $\phi_{3}=0$, $Y_{t}=\phi_{1}^{\left(t\right)}\circ Y_{t-1}+\phi_{3}^{\left(t\right)}\circ Y_{t-3}+Z_{t}$ represents an incorrect model setting and the correct model setting, in this case, should be $Y_{t}=\phi_{1}^{\left(t\right)}\circ Y_{t-1}+Z_{t}$. As shown in Figure 2, when the sample size is small, particularly when the sample size is 100, the performance of $P_{T}=T^{\frac{1}{5}}$ is notably improved compared to $P_{T}=log(T)$ and $P_{T}=T^{\frac{1}{3}}$. As the sample size increases, this advantage gradually diminishes, but the penalty term setting $P_{T}=T^{\frac{1}{5}}$ still maintains an advantage when $\phi_{3}$ is relatively close to 0.

Based on the numerical simulation results presented in this subsection, we can offer recommendations for applying the penalized criteria (6): when the sample size is small, or some coefficients in the true model are relatively close to 0, we can employ the penalty term setting $P_{T}=T^{\frac{1}{5}}$. In other cases, the performance of the penalty term settings $P_{T}=T^{\frac{1}{3}}$ and $P_{T}=log(T)$ is comparable and slightly better than $P_{T}=T^{\frac{1}{5}}$. Furthermore, we also conducted a simulation study on lag variable selection for the data-generating process:

$$Y_{t}=\phi_{2}^{\left(t\right)}\circ Y_{t-2}+Z_{t}$$

where the mean of $\phi_{2}^{\left(t\right)}$ is 0.3. The results can be found in Table A1 in Appendix A.

### 3.2. Performance of Penalized Criteria in INARCH Models and AR Models

As stated in the Remark of Section 2, we can apply the penalty criteria (6) to both INARCH and AR models. Because the likelihood functions for these two models can be easily established, we can compare the performance of the penalty criteria (6) with that of AIC and BIC for both these models.

#### 3.2.1. INARCH Model

In this subsection, we consider the true data-generating process as follows:

$$Y_{t}|F_{t-1}\sim Poisson(\lambda_{t})$$

$$\lambda_{t}=\phi_{0}+\phi_{1}Y_{t-1}+\phi_{3}Y_{t-3}$$

where $\phi_{1}=0.4$, $\phi_{3}=0.2$, and $\phi_{0}=2$. Fokianos, Rahbek, and Tjøstheim [26] proposed this model and derived the conditions for its stationarity and ergodicity. By applying the penalized criteria (6) alongside AIC and BIC, we attempt to select the true model from all INARCH models up to the third order. In Table 2:

i.i.d. represents an *i.i.d. Poisson* random variable sequence,
$y_{t-1}$ represents the model $Y_{t}|F_{t-1}\sim Poisson\left(\lambda_{t}\right)\lambda_{t}=\phi_{0}+\phi_{1}Y_{t-1}$,
$y_{t-2}\;represents\;the\;model\;Y_{t}|F_{t-1}\sim Poisson\left(\lambda_{t}\right)\lambda_{t}=\phi_{0}+\phi_{2}Y_{t-2}$,
$y_{t-3}\;represents\;the\;model\;Y_{t}|F_{t-1}\sim Poisson\left(\lambda_{t}\right)\lambda_{t}=\phi_{0}+\phi_{3}Y_{t-3}$,
$y_{t-1}{,y}_{t-2}\;represents\;the\;model\;Y_{t}|F_{t-1}\sim Poisson\left(\lambda_{t}\right)\lambda_{t}=\phi_{0}+\phi_{1}Y_{t-1}+\phi_{2}Y_{t-2}$,
$y_{t-1}{,y}_{t-3}\;represents\;the\;model\;Y_{t}|F_{t-1}\sim Poisson\left(\lambda_{t}\right)\lambda_{t}=\phi_{0}+\phi_{1}Y_{t-1}+\phi_{3}Y_{t-3}$,
$y_{t-1}{,y}_{t-3}\;represents\;the\;model\;Y_{t}|F_{t-1}\sim Poisson\left(\lambda_{t}\right)\lambda_{t}=\phi_{0}+\phi_{2}Y_{t-2}+\phi_{3}Y_{t-3}$,
$y_{t-1}{,y}_{t-2}{,y}_{t-3}\;represents\;the\;model\;Y_{t}|F_{t-1}\sim Poisson\left(\lambda_{t}\right)$,
  $$\lambda_{t}=\phi_{0}+\phi_{1}Y_{t-1}+\phi_{2}Y_{t-2}+\phi_{3}Y_{t-3}.$$
  “Criterion” denotes the model selection criteria we use, and we use $H+P_{T}\cdot \left|m\right|$ to denote penalized criteria (6). Furthermore, we have bolded the true model $y_{t-1}{,y}_{t-3}$. We consider sample sizes *T* = 100, 200, 300, 500, 1000, and for each sample size *T* and parameter setting, we conduct 1000 independent repeated experiments.

From Table 2, we can observe that, similar to the INAR case, the accuracy of $P_{T}=T^{\frac{1}{5}}$ is slightly worse than $P_{T}=T^{\frac{1}{3}}$ and $P_{T}=log(T)$ in larger sample sizes, but in smaller sample sizes, i.e., T≤500, the performance of $P_{T}=T^{\frac{1}{5}}$ is superior. In addition, from Table 2, we can observe that the accuracy of the penalized criteria proposed in this paper is roughly equivalent to BIC when $P_{T}=T^{\frac{1}{3}}$ and $P_{T}=log(T)$, while the accuracy is roughly equivalent to AIC in small samples when $P_{T}=T^{\frac{1}{5}}$, but $P_{T}=T^{\frac{1}{5}}$ is far better than AIC when the sample size is large.

**Table 2:** Frequency of model selection for INARCH model of order 2 by the penalized criterion (6).

$Y_{t}|F_{t-1}\sim Poisson(\lambda_{t})$										
$\lambda_{t}=\phi_{0}+\phi_{1}Y_{t-1}+\phi_{3}Y_{t-3}$										
$\phi_{1}=0.4$ $,\;\phi_{3}=0.2$			Models to Be Selected							
T	Criterion	$P_{T}$	i.i.d.	$y_{t-1}$	$y_{t-2}$	$y_{t-3}$	$y_{t-1}{,y}_{t-2}$	$y_{t-1}{,y}_{t-3}$	$y_{t-2}{,y}_{t-3}$	$y_{t-1}{,y}_{t-2}{,y}_{t-3}$
100	$H+P_{T}\cdot \left|m\right|$	$T^{1/3}$	0.059	0.576	0.001	0.019	0.021	**0.309**	0.001	0.014
		log(T)	0.057	0.568	0.001	0.017	0.021	**0.32**	0.001	0.015
		$T^{1/5}$	0.012	0.337	0	0.007	0.054	**0.509**	0.003	0.078
	AIC		0.003	0.27	0	0.006	0.054	**0.559**	0.001	0.107
	BIC		0.032	0.551	0.002	0.021	0.022	**0.361**	0.001	0.01
200	$H+P_{T}\cdot \left|m\right|$	$T^{1/3}$	0	0.406	0	0.002	0.013	**0.574**	0	0.005
		log(T)	0	0.359	0	0.001	0.017	**0.612**	0	0.011
		$T^{1/5}$	0	0.138	0	0	0.028	**0.756**	0	0.078
	AIC		0	0.068	0	0	0.024	**0.774**	0	0.134
	BIC		0	0.296	0	0.001	0.019	**0.673**	0	0.011
300	$H+P_{T}\cdot \left|m\right|$	$T^{1/3}$	0	0.22	0	0	0.07	**0.767**	0	0.006
		log(T)	0	0.153	0	0	0.011	**0.826**	0	0.01
		$T^{1/5}$	0	0.041	0	0	0.01	**0.874**	0	0.075
	AIC		0	0.016	0	0	0.006	**0.832**	0	0.146
	BIC		0	0.127	0	0	0.008	**0.855**	0	0.01
500	$H+P_{T}\cdot \left|m\right|$	$T^{1/3}$	0	0.035	0	0	0.03	**0.958**	0	0.004
		log(T)	0	0.017	0	0	0.002	**0.971**	0	0.01
		$T^{1/5}$	0	0.001	0	0	0.002	**0.934**	0	0.063
	AIC		0	0	0	0	0.001	**0.841**	0	0.158
	BIC		0	0.012	0	0	0.004	**0.976**	0	0.008
1000	$H+P_{T}\cdot \left|m\right|$	$T^{1/3}$	0	0	0	0	0	**1**	0	0
		log(T)	0	0	0	0	0	**0.991**	0	0.009
		$T^{1/5}$	0	0	0	0	0	**0.956**	0	0.044
	AIC		0	0	0	0	0	**0.848**	0	0.152
	BIC		0	0	0	0	0	**0.995**	0	0.005

Additionally, we provide a simulation study on lag variable selection for the data-generating process:

$$Y_{t}|F_{t-1}\sim Poisson(\lambda_{t})$$

$$\lambda_{t}=\phi_{0}+\phi_{1}Y_{t-1}.$$

The results can be found in Table A2 in the Appendix A.

#### 3.2.2. AR Model

In this subsection, we consider the true data-generating process as follows:

(8) $$Y_{t}=\phi_{0}+\phi_{1}Y_{t-1}+\phi_{3}Y_{t-3}+Z_{t}$$

where $\phi_{1}=0.4$, $\phi_{3}=0.2$, $\phi_{0}=1$, and $Z_{t}$ follows a normal distribution with a mean of 0 and a standard deviation of 2. By applying the penalized criteria (6) alongside AIC and BIC, we attempt to select the true model from all AR models up to the third order. In Table 3:

i.i.d. represents an i.i.d. Normal random variable sequence,
$y_{t-1}$ represents the model $Y_{t}=\phi_{0}+\phi_{1}Y_{t-1}+Z_{t}$,
$y_{t-2}\;represents\;the\;model\;Y_{t}=\phi_{0}+\phi_{2}Y_{t-2}+Z_{t}$,
$y_{t-3}\;represents\;the\;model\;Y_{t}=\phi_{0}+\phi_{3}Y_{t-3}+Z_{t}$,
$y_{t-1}{,y}_{t-2}\;represents\;the\;model\;Y_{t}=\phi_{0}+\phi_{1}Y_{t-1}+\phi_{2}Y_{t-2}+Z_{t}$,
$y_{t-1}{,y}_{t-3}\;represents\;the\;model\;Y_{t}=\phi_{0}+\phi_{1}Y_{t-1}+\phi_{3}Y_{t-3}+Z_{t}$,
$y_{t-1}{,y}_{t-3}\;represents\;the\;model\;Y_{t}=\phi_{0}+\phi_{2}Y_{t-2}+\phi_{3}Y_{t-3}+Z_{t}$,
$y_{t-1}{,y}_{t-2}{,y}_{t-3}\;represents\;the\;model\;Y_{t}=\phi_{0}+\phi_{1}Y_{t-1}+\phi_{2}Y_{t-2}+\phi_{3}Y_{t-3}+Z_{t}$,

“Criterion” denotes the model selection criteria we use, and we use $H+P_{T}\cdot \left|m\right|$ to denote penalized criteria (6). We use boldface to highlight the true model:

**Table 3:** Frequency of model selection AR model of order 1 by the penalized criterion (6).

$Y_{t}=\phi_{0}+\phi_{1}Y_{t-1}+\phi_{3}Y_{t-3}+Z_{t}$										
$\phi_{1}=0.4$ $,\;\phi_{3}=0.2$			Models to Be Selected							
T	Criterion	$P_{T}$	i.i.d.	$y_{t-1}$	$y_{t-2}$	$y_{t-3}$	$y_{t-1}{,y}_{t-2}$	$y_{t-1}{,y}_{t-3}$	$y_{t-2}{,y}_{t-3}$	$y_{t-1}{,y}_{t-2}{,y}_{t-3}$
100	$H+P_{T}\cdot \left|m\right|$	$T^{1/3}$	0.048	0.577	0.002	0.02	0.018	**0.324**	0.001	0.01
		log(T)	0.048	0.564	0.002	0.02	0.018	**0.336**	0.001	0.011
		$T^{1/5}$	0.009	0.34	0.002	0.012	0.044	**0.524**	0.001	0.068
	AIC		0.004	0.255	0.002	0.005	0.059	**0.578**	0.002	0.095
	BIC		0.032	0.564	0.001	0.016	0.018	**0.352**	0.001	0.016
200	$H+P_{T}\cdot \left|m\right|$	$T^{1/3}$	0.001	0.323	0	0.001	0.011	**0.654**	0	0.01
		log(T)	0.001	0.279	0	0	0.014	**0.693**	0	0.013
		$T^{1/5}$	0	0.11	0	0	0.025	**0.79**	0	0.075
	AIC		0	0.062	0	0	0.021	**0.771**	0	0.146
	BIC		0.001	0.261	0	0	0.013	**0.713**	0	0.012
300	$H+P_{T}\cdot \left|m\right|$	$T^{1/3}$	0	0.167	0	0	0.004	**0.825**	0	0.004
		log(T)	0	0.116	0	0	0.004	**0.874**	0	0.006
		$T^{1/5}$	0	0.042	0	0	0.007	**0.893**	0	0.058
	AIC		0	0.017	0	0	0.007	**0.824**	0	0.152
	BIC		0	0.107	0	0	0.005	**0.88**	0	0.008
500	$H+P_{T}\cdot \left|m\right|$	$T^{1/3}$	0	0.034	0	0	0.003	**0.959**	0	0.004
		log(T)	0	0.013	0	0	0.004	**0.975**	0	0.008
		$T^{1/5}$	0	0.002	0	0	0.001	**0.937**	0	0.06
	AIC		0	0	0	0	0	**0.837**	0	0.163
	BIC		0	0.011	0	0	0.003	**0.977**	0	0.009
1000	$H+P_{T}\cdot \left|m\right|$	$T^{1/3}$	0	0	0	0	0	**0.996**	0	0.004
		log(T)	0	0	0	0	0	**0.989**	0	0.011
		$T^{1/5}$	0	0	0	0	0	**0.951**	0	0.046
	AIC		0	0	0	0	0	**0.846**	0	0.154
	BIC		0	0	0	0	0	**0.988**	0	0.012

From Table 3, we can observe that, similar to the INAR case, the accuracy of $P_{T}=T^{\frac{1}{5}}$ is slightly worse than $P_{T}=T^{\frac{1}{3}}$ and $P_{T}=log(T)$ in larger sample sizes, but in smaller sample sizes, i.e., T≤500, the performance of $P_{T}=T^{\frac{1}{5}}$ is superior. The comparison of the penalized criteria proposed in this paper with AIC and BIC in the AR model is analogous to that in the INARCH model; thus further elaboration is not required.

### 3.3. Robustness of Variable Selection Procedure

In this section, we investigate the robustness of the penalized criteria (6) for different distributions of the innovation term $Z_{t}$ in model (7). Specifically, we consider $Z_{t}$ to follow a Poisson distribution, a geometric distribution with a mean of 2, and a uniform distribution over {0,1,2,3,4}. In Table 4, “$Z_{t}$” denotes the random distribution of the innovation term, whereas “geom” denotes the geometric distribution.

Through Table 4, we observe that the penalized criteria (6) remain robust for various distributions of the innovation term $Z_{t}$. This finding suggests that the criteria proposed in this paper can effectively select the correct lag order even when the innovation term adheres to different distributions. We use boldface to highlight the true model:

**Table 4:** Frequency of model selection of INAR model by the penalized criterion (6) with $Z_{t}$ misspecification.

$Y_{t}=\phi_{1}^{\left(t\right)}\circ Y_{t-1}+\phi_{3}^{\left(t\right)}\circ Y_{t-3}+Z_{t}$										
$\phi_{1}=0.4,\phi_{3}=0.2$			Models to Be Selected							
T	$Z_{t}$	$P_{T}$	i.i.d.	$y_{t-1}$	$y_{t-2}$	$y_{t-3}$	$y_{t-1}{,y}_{t-2}$	$y_{t-1}{,y}_{t-3}$	$y_{t-2}{,y}_{t-3}$	$y_{t-1}{,y}_{t-2}{,y}_{t-3}$
100	Poisson	$T^{1/3}$	0.045	0.587	0.003	0.018	0.018	**0.319**	0	0.01
		log(T)	0.043	0.584	0.003	0.019	0.018	**0.323**	0	0.01
		$T^{1/5}$	0.005	0.349	0.002	0.008	0.053	**0.514**	0.003	0.066
	Uniform	$T^{1/3}$	0.048	0.564	0.001	0.02	0.018	**0.336**	0.001	0.048
		log(T)	0.043	0.559	0	0.019	0.02	**0.345**	0.001	0.043
		$T^{1/5}$	0.014	0.332	0	0.004	0.047	**0.519**	0	0.014
	Geom	$T^{1/3}$	0.07	0.575	0.004	0.032	0.023	**0.285**	0.001	0.02
		log(T)	0.067	0.57	0.004	0.032	0.024	**0.292**	0.001	0.02
		$T^{1/5}$	0.009	0.327	0.002	0.011	0.052	**0.511**	0.002	0.086
200	Poisson	$T^{1/3}$	0	0.37	0	0.001	0.008	**0.612**	0	0.008
		log(T)	0	0.319	0	0.002	0.012	**0.655**	0	0.012
		$T^{1/5}$	0	0.109	0	0.001	0.02	**0.795**	0	0.075
	Uniform	$T^{1/3}$	0	0.343	0	0	0.012	**0.636**	0	0.009
		log(T)	0	0.29	0	0	0.016	**0.681**	0	0.013
		$T^{1/5}$	0	0.13	0	0	0.026	**0.776**	0	0.068
	Geom	$T^{1/3}$	0.005	0.358	0	0	0.018	**0.603**	0	0.016
		log(T)	0.004	0.312	0	0	0.02	**0.643**	0	0.021
		$T^{1/5}$	0	0.108	0	0	0.034	**0.752**	0	0.106
300	Poisson	$T^{1/3}$	0	0.193	0	0	0.003	**0.801**	0	0.003
		log(T)	0	0.138	0	0	0.004	**0.852**	0	0.006
		$T^{1/5}$	0	0.044	0	0	0.005	**0.878**	0	0.073
	Uniform	$T^{1/3}$	0	0.184	0	0	0.01	**0.802**	0	0.004
		log(T)	0	0.122	0	0	0.012	**0.851**	0	0.015
		$T^{1/5}$	0	0.03	0	0	0.011	**0.885**	0	0.074
	Geom	$T^{1/3}$	0	0.188	0	0	0.012	**0.796**	0	0.004
		log(T)	0	0.133	0	0	0.015	**0.834**	0	0.018
		$T^{1/5}$	0	0.027	0	0	0.013	**0.88**	0	0.08
500	Poisson	$T^{1/3}$	0	0.027	0	0	0.005	**0.962**	0	0.006
		log(T)	0	0.008	0	0	0.005	**0.975**	0	0.012
		$T^{1/5}$	0	0.002	0	0	0.003	**0.923**	0	0.072
	Uniform	$T^{1/3}$	0	0.04	0	0	0.004	**0.95**	0	0.006
		log(T)	0	0.02	0	0	0.002	**0.964**	0	0.014
		$T^{1/5}$	0	0.003	0	0	0	**0.926**	0	0.071
	Geom	$T^{1/3}$	0	0.035	0	0	0.008	**0.954**	0	0.003
		log(T)	0	0.018	0	0	0.007	**0.964**	0	0.011
		$T^{1/5}$	0	0.002	0	0	0.003	**0.928**	0	0.067
1000	Poisson	$T^{1/3}$	0	0	0	0	0	**0.998**	0	0.002
		log(T)	0	0	0	0	0	**0.989**	0	0.011
		$T^{1/5}$	0	0	0	0	0	**0.952**	0	0.048
	Uniform	$T^{1/3}$	0	0	0	0	0	**1**	0	0
		log(T)	0	0	0	0	0	**0.995**	0	0.05
		$T^{1/5}$	0	0	0	0	0	**0.944**	0	0.056
	Geom	$T^{1/3}$	0	0	0	0	0	**0.994**	0	0.006
		log(T)	0	0	0	0	0	**0.982**	0	0.018
		$T^{1/5}$	0	0	0	0	0	**0.947**	0	0.053

Furthermore, we compare the performance of the penalized criteria proposed in this paper, AIC, and BIC when the innovation term $Z_{t}$ in AR model (8) follows a uniform distribution over [−2, 2] while the assumption of $Z_{t}$ is a normal distribution with mean 0 and unknown variance $\sigma_{Z}^{2}$. In Appendix A, Table A3 shows that regardless of the distribution of the innovation term, when the conditional mean is set correctly, the performance and robustness of the penalized criteria proposed in this paper are generally equivalent to those of AIC and BIC.

## 4. Real Data Application

### 4.1. COVID-19 Infection Data

**Figure 3:**
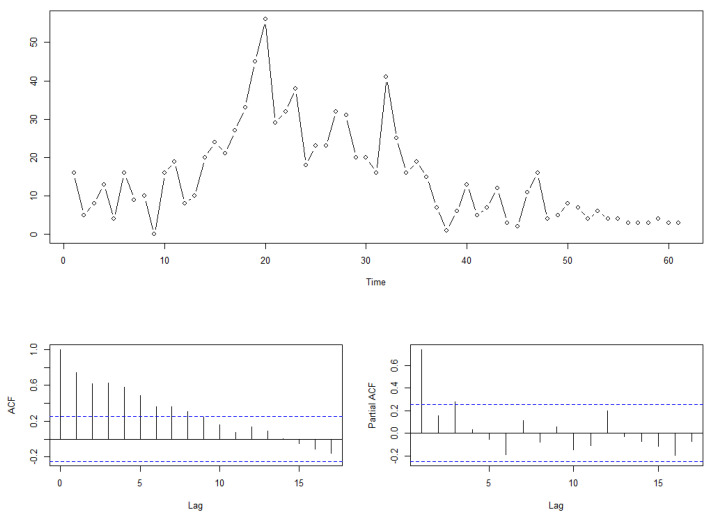
Number of COVID-19 infections in Cyprus, 13 March to 12 May 2020.

The investigation of data related to infectious diseases constitutes a crucial application of integer-valued time series models within the public health domain. In May 2020, the Ministry of Health in Cyprus disseminated a national epidemic surveillance report, which displayed the temporal data pertaining to the number of infections during the initial phase of the COVID-19 outbreak. Conducting research on this data is instrumental for the public health academia in uncovering the intrinsic mechanisms governing epidemic propagation. Owing to the incubation period associated with the coronavirus, individuals who contract the virus typically disclose their infection status to governmental statistical departments after a lapse of several days. As a result, it becomes imperative to scrutinize the matter of lag variable selection within this time series dataset, see Figure 3 below.

Based on the ACF plot, it can be inferred that the data may stem from an autoregressive data-generating process. The PACF plot suggests that selecting either the model:

$$Y_{t}=\phi_{1}^{\left(t\right)}\circ Y_{t-1}+Z_{t}$$

or

$$Y_{t}=\phi_{1}^{\left(t\right)}\circ Y_{t-1}+\phi_{3}^{\left(t\right)}\circ Y_{t-3}+Z_{t}$$

is reasonable, as the partial autocorrelation function for a lag of three periods does not significantly exceed the critical value. Consequently, we employ the model selection procedure (6) for variable selection, and we provide the result in the following Table 5.

Given that the penalized criteria (6) favor the model

$$Y_{t}=\phi_{1}^{\left(t\right)}\circ Y_{t-1}+\phi_{3}^{\left(t\right)}\circ Y_{t-3}+Z_{t}$$

under all three penalty settings, we adopt this model. The estimated results for this model are:

$$\begin{array}{llll} Y_{t}= & 0.5736\circ Y_{t-1} & + & 0.2933\circ Y_{t-3}+Z_{t}\\ & \left(0.1081\right) & & \left(0.1092\right) \end{array}$$

where the mean of $Z_{t}$ is 1.8567; $\phi_{1}^{\left(t\right)}$ and $\phi_{3}^{\left(t\right)}$ as two non-negative random variables, have expected values of 0.5736 and 0.2933, respectively. This finding suggests that during the initial stages of the outbreak in Cyprus, the number of infections on a given day may have been influenced by the number of infections one day and three days prior.

### 4.2. Seismic Frequency Data

**Figure 4:**
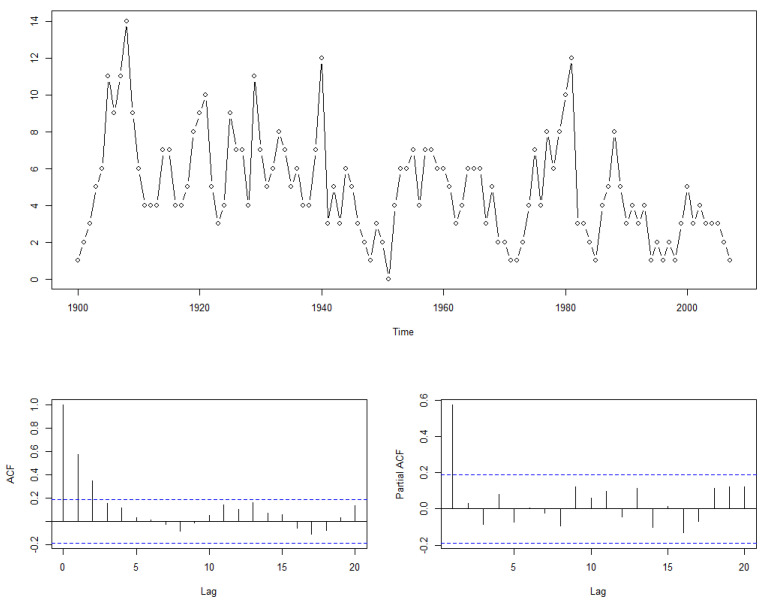
Global frequency of earthquakes of magnitude seven or greater between 1900 and 2007.

The exploration of earthquake frequency constitutes a significant application frontier for integer-valued time series models. As documented by Zucchini, MacDonald, and Langrock [27], comprehensive annual data delineating global seismic occurrences of magnitude seven or above, encompassing the period from 1900 to 2007, has been provided. This wealth of data offers a promising platform for scholars seeking to unravel the intricate mechanisms underpinning the mutual interactions among earthquakes. It is envisaged that through a meticulous investigation of this time-series data associated with seismic activities, one might gain insights into whether the interplay is mediated by crustal stress dynamics or alternative conduits, see Figure 4 below.

Informed by the ACF, we hypothesize that the underlying data generation process might be suitably modeled by an autoregressive construct. On the other hand, insights gleaned from the PACF advocate for the application of a first-order autoregressive model. To substantiate this conjecture further, we will proceed to invoke the penalized criterion (6) as our analytical tool in the ensuing discourse, and we provide the result in the following Table 6.

Given that under the three penalty settings, the penalized criterion (6) exhibits a preference for the model

$$Y_{t}=\phi_{1}^{\left(t\right)}\circ Y_{t-1}+Z_{t}$$

we opt to adopt this model. The estimated results for this model are as follows:

$$Y_{t}=0.5799\circ Y_{t-1}+Z_{t}\;\left(0.0812\right)$$

In this model, the mean value of $Z_{t}$ is identified as 2.1014. The derived estimations posit that every occurrence of a magnitude seven or higher earthquake in the preceding year induces a count of similar-intensity earthquakes in the subsequent year, which manifests as a discrete random variable with an expected value of 0.5799. Simultaneously, the number of major earthquakes occurring independently each year is approximately two. These results substantiate the existence of a year-on-year time-varying dependency mechanism in the frequency of major seismic disasters.

## 5. Discussion and Conclusions

In this paper, we propose a model selection criterion based on an estimation equation established in Conditional Least Squares estimation. This penalized method does not rely on detailed distributional assumptions for the data-generating process. It circumvents the complex likelihood function construction in Random Coefficient Integer-Valued Autoregressive models and can consistently select the correct variables under relatively mild assumptions.

In our numerical simulations, we compared the impact of three penalty term settings on the performance of the penalty criteria. We found that the impact of these penalty terms on the performance of the information criteria varies as partial coefficients in the RCINAR model move farther away from 0 or as the sample size increases. Moreover, we applied the model selection method proposed in this paper to both the INARCH and traditional continuous-valued AR models. We discovered that in both scenarios where likelihood functions can be easily constructed, the proposed model selection criteria and the traditional likelihood-based information criteria, AIC and BIC, exhibit similar model selection efficiency. Specifically, under the settings of $P_{T}=T^{\frac{1}{3}}$ and $P_{T}=log(T)$, the accuracy of the proposed model selection method is similar to that of BIC. However, in cases with smaller sample sizes, the proposed model selection method with $P_{T}=T^{\frac{1}{5}}$ performs similarly to AIC while outperforming AIC with larger sample sizes.

In the future, model selection methods based on estimation equations have considerable potential for development. In this discussion section, we briefly introduce three aspects:

(1) Distinguishing between different thinning operators or innovation terms with varying distributions: The criterion (6) provided in this paper is primarily used for lag variable selection but lacks the ability to differentiate between various thinning operators and distinct distributions of innovation terms. It is well known that INAR models can describe scenarios such as zero inflation, variance inflation, and extreme values by flexibly selecting thinning operators and innovation terms. Therefore, if a model selection criterion can distinguish between different thinning operators and varying distributions of innovation terms, it will have a more extensive application scope.
(2) Incorporating higher-order conditional moments from the data-generating process into the information criterion. Through the form of the $H(\tilde{\theta })$ function:
  $$H\left(\tilde{\theta }\right)={\left(\sum_{t=p+1}^{T}\Psi_{t}\left(\tilde{\theta }\right)\right)}^{\prime }{\left(\sum_{t=p+1}^{T}\Psi_{t}\left(\tilde{\theta }\right)\Psi_{t}{\left(\tilde{\theta }\right)}^{\prime }\right)}^{-1}\left(\sum_{t=p+1}^{T}\Psi_{t}\left(\tilde{\theta }\right)\right)$$
  It is evident that criterion (6) only contains the mean structure information of the model and lacks the ability to describe higher-order moment information. Since many variants of the INAR model exhibit differences in higher-order moments, incorporating higher-order moment information into the model selection criterion would enable criterion (6) to perform model selection within a broader context.
(3) Detecting change points. In the field of time series data research, the change point detection problem has a long history. Specifically, within the integer-valued time series domain, the change point problem refers to the existence of positive integers $\tau_{1},\tau_{2},\cdots ,\tau_{m}$, such that:
  $$Y_{t}=\left\{\right.\begin{array}{cc} \phi^{\left(1\right)}\circ Y_{\left\{t-1\right)}+Z_{t}^{\left(1\right)} & 0<t\leq \tau_{1}\\ \phi^{\left(2\right)}\circ Y_{\left\{t-1\right)}+Z_{t}^{\left(2\right)} & \tau_{1}<t\leq \tau_{2}\\ \vdots & \vdots \\ \phi^{\left(m\right)}\circ Y_{\left\{t-1\right)}+Z_{t}^{\left(m\right)} & \tau_{m}<t\leq T \end{array}$$
  For continuous-valued time series models, Chen and Gupta [28] introduced a method for change point detection using AIC and BIC. Since parameter changes are prominently reflected in the mean structure of INAR models, it is likely feasible to perform change point detection using the criterion (6) based on the estimation equations.

## Figures and Tables

**Table 5:** Result of model selection with COVID-19 data.

$P_{T}$	i.i.d.	$y_{t-1}$	$y_{t-2}$	$y_{t-3}$	$y_{t-1}{,y}_{t-2}$	$y_{t-1}{,y}_{t-3}$	$y_{t-2}{,y}_{t-3}$	$y_{t-1}{,y}_{t-2}{,y}_{t-3}$
$T^{1/3}$	28.6	15.9	22.9	20.5	17.9	**11.8**	22.9	15.7
log(T)	28.7	16.3	23.3	20.8	18.4	**12.3**	23.4	16.4
$T^{1/5}$	26.9	12.6	19.6	17.1	12.9	**6.8**	17.9	9.1

**Table 6:** Result of model selection with seismic frequency data.

$P_{T}$	i.i.d.	$y_{t-1}$	$y_{t-2}$	$y_{t-3}$	$y_{t-1}{,y}_{t-2}$	$y_{t-1}{,y}_{t-3}$	$y_{t-2}{,y}_{t-3}$	$y_{t-1}{,y}_{t-2}{,y}_{t-3}$
$T^{1/3}$	29.8	**10.7**	29.5	35.9	15.3	15.1	33.1	19.0
log(T)	29.7	**10.6**	29.3	35.8	15.0	12.9	32.8	18.7
$T^{1/5}$	27.5	**6.3**	25.1	31.6	8.6	8.5	26.5	10.2

## Data Availability

The data has been uploaded as a Supplementary File of this paper. Interested readers are also encouraged to request the relevant data and code from the authors directly through e-mail.

## Supplementary Materials

The following supporting information can be downloaded at: https://www.mdpi.com/article/10.3390/e25081220/s1.

## Appendix A

### Appendix A.1. Proofs

**Proof** **of** **Lemma** **1.**

(1) From Lemma 1 in Zhang, Wang, and Zhu [7], it can be proved immediately.
(2) Due to the construction of $\Psi_{t}\left(\theta \right)$, we have $\frac{\partial^{2}\Psi_{t}\left(\theta \right)}{\partial \theta \partial \theta^{\prime }}=0$, which proves the statement.
(3) Because the construction of $\Psi_{t}\left(\theta \right)$ ensures that $\frac{\partial \Psi_{t}\left(\theta \right)}{\partial \theta^{\prime }}$ is a constant with respect to the parameter vector θ and is only related to $\left\{Y_{t}\right\}$, the conclusion holds under Assumption (A1). □

**Proof** **of** **Theorem** **1.** When model m is correctly specified, by Taylor expansion, we have:

$$\sum_{t=p+1}^{T}\Psi_{t}\left({\hat{\theta }}_{CLS}\left(m\right)\right)=\sum_{t=p+1}^{T}\Psi_{t}\left({\tilde{\theta }}_{0}\right)+\sum_{t=p+1}^{T}\frac{\partial \Psi_{t}\left({\tilde{\theta }}_{0}\right)}{\partial {\tilde{\theta }}^{\prime }}\left({\hat{\theta }}_{CLS}\left(m\right)-{\tilde{\theta }}_{0}\right)+o_{p}\left(T^{-\frac{1}{2}}\right)$$

where, according to Equation (5), $o_{p}\left(\left\|{\hat{\theta }}_{CLS}\left(m\right)-{\tilde{\theta }}_{0}\right\|\right)=o_{p}\left(T^{-\frac{1}{2}}\right)$. Since ${\hat{\theta }}_{\left(2\right),CLS}^{}\left(m\right)={\tilde{\theta }}_{\left(2\right)}\left(m\right)=0$, then:

$$0=\frac{1}{T}\sum_{t=p+1}^{T}\Psi_{1t}\left({\hat{\theta }}_{CLS}\left(m\right)\right)\;=\frac{1}{T}\sum_{t=p+1}^{T}\Psi_{1t}\left({\tilde{\theta }}_{0}\right)+\frac{1}{T}\sum_{t=p+1}^{T}\frac{\partial \Psi_{t}\left({\tilde{\theta }}_{0}\right)}{\partial \theta_{\left(1\right)}^{\prime }\left(m\right)}\left({\hat{\theta }}_{\left(1\right),CLS}^{}\left(m\right)-{\tilde{\theta }}_{\left(1\right)}\left(m_{0}\right)\right)+o_{p}\left(T^{-\frac{1}{2}}\right)$$

Thus, we have:

$${\hat{\theta }}_{\left(1\right),CLS}^{}\left(m\right)-{\tilde{\theta }}_{\left(1\right)}\left(m_{0}\right)=-{\left(\frac{1}{T}\sum_{t=p+1}^{T}\frac{\partial \Psi_{t}\left({\tilde{\theta }}_{0}\right)}{\partial {\tilde{\theta }}_{\left(1\right)}^{\prime }\left(m\right)}\right)}^{-1}\frac{1}{T}\sum_{t=p+1}^{T}\Psi_{1t}\left({\tilde{\theta }}_{0}\right)+o_{p}\left(T^{-\frac{1}{2}}\right)\;{=-\left(E\left(\frac{\partial \Psi_{t}\left({\tilde{\theta }}_{0}\right)}{\partial {\tilde{\theta }}_{\left(1\right)}^{\prime }\left(m\right)}\right)\right)}^{-1}\frac{1}{T}\sum_{t=p+1}^{T}\Psi_{1t}\left({\tilde{\theta }}_{0}\right)+o_{p}\left(T^{-\frac{1}{2}}\right)$$

Therefore:

$${\hat{\theta }}_{CLS}\left(m\right)-{\tilde{\theta }}_{0}=\left[\begin{array}{c} {\hat{\theta }}_{\left(1\right),CLS}^{}\left(m\right)-{\tilde{\theta }}_{\left(1\right)}\left(m_{0}\right)\\ 0 \end{array}\right]\;=\left[\begin{array}{c} -{\left(E\left(\frac{\partial \Psi_{t}\left({\tilde{\theta }}_{0}\right)}{\partial {\tilde{\theta }}_{\left(1\right)}^{\prime }\left(m\right)}\right)\right)}^{-1}\frac{1}{T}\sum_{t=p+1}^{T}\Psi_{1t}\left({\tilde{\theta }}_{0}\right)+o_{p}\left(T^{-\frac{1}{2}}\right)\\ 0 \end{array}\right]$$

Hence:

$$\frac{1}{T}\sum_{t=p+1}^{T}\Psi_{t}\left({\hat{\theta }}_{CLS}\left(m\right)\right)\;=\left(I-E\left(\frac{\partial \Psi_{t}\left({\tilde{\theta }}_{0}\right)}{\partial {\tilde{\theta }}^{\prime }}\right)\left[\begin{array}{cc} {\left(E\left(\frac{\partial \Psi_{t}\left({\tilde{\theta }}_{0}\right)}{\partial {\tilde{\theta }}_{\left(1\right)}^{\prime }\left(m\right)}\right)\right)}^{-1} & 0\\ 0 & 0 \end{array}\right]\right)\frac{1}{T}\sum_{t=p+1}^{T}\Psi_{t}\left({\tilde{\theta }}_{0}\right)+o_{p}\left(T^{-\frac{1}{2}}\right)\;≝\Sigma_{*}\frac{1}{T}\sum_{t=p+1}^{T}\Psi_{t}\left({\tilde{\theta }}_{0}\right)+op\left(T^{-\frac{1}{2}}\right)$$

where I is the identity matrix, and let $\Sigma_{11}=E\left(\Psi_{t}\left({\tilde{\theta }}_{0}\right)\Psi_{t}{\left({\tilde{\theta }}_{0}\right)}^{\prime }\right)$, then by Lemma 1, we have:

$$H\left({\hat{\theta }}_{CLS}\left(m\right)\right)={\left(\sum_{t=p+1}^{T}\Psi_{t}\left({\hat{\theta }}_{CLS}\left(m\right)\right)\right)}^{\prime }{\left(\sum_{t=p+1}^{T}\Psi_{t}\left({\hat{\theta }}_{CLS}\left(m\right)\right)\Psi_{t}{\left({\hat{\theta }}_{CLS}\left(m\right)\right)}^{\prime }\right)}^{-1}\left(\sum_{t=p+1}^{T}\Psi_{t}\left({\hat{\theta }}_{CLS}\left(m\right)\right)\right)\;={\left(T^{-\frac{1}{2}}\sum_{t=p+1}^{T}\Sigma_{11}^{-\frac{1}{2}}\Psi_{t}\left({\tilde{\theta }}_{0}\right)\right)}^{\prime }\Sigma_{11}^{\frac{1}{2}}\Sigma_{*}^{\prime }\Sigma_{11}^{-1}\Sigma_{*}\Sigma_{11}^{\frac{1}{2}}\left(T^{-\frac{1}{2}}\sum_{t=p+1}^{T}\Sigma_{11}^{-\frac{1}{2}}\Psi_{t}\left({\tilde{\theta }}_{0}\right)\right)+O_{p}(1)$$

Let $\Omega =\Sigma_{11}^{\frac{1}{2}}\Sigma_{*}^{\prime }\Sigma_{11}^{-1}\Sigma_{*}\Sigma_{11}^{\frac{1}{2}}=\Sigma_{11}^{\frac{1}{2}}\Sigma_{*}^{\prime }\Sigma_{11}^{-\frac{1}{2}}{\Sigma_{11}^{-\frac{1}{2}}\Sigma }_{*}\Sigma_{11}^{\frac{1}{2}}$, which implies that Ω is a positive semi-definite matrix. Consequently, according to Johnston [29]’s Theorem 2.1.6, we have ${\Omega =U\Lambda U}^{\prime }$, where U is an orthogonal matrix, Λ is a diagonal matrix, and the diagonal elements of Λ are the eigenvalues of Ω, denoted as $\Lambda_{1},\ldots ,\Lambda_{p+1}$. Thus,

$$H\left({\hat{\theta }}_{CLS}\left(m\right)\right)=\sum_{j=1}^{p+1}\Lambda_{j}{\left[T^{-\frac{1}{2}}\sum_{t=p+1}^{T}\Sigma_{11}^{-\frac{1}{2}}\Psi_{t}\left({\tilde{\theta }}_{0}\right)\right]}_{j}^{2}+O_{p}(1)$$

where ${\left[J\right]}_{j}$ denotes the j-th element of vector J. Therefore, we obtain:

$$H\left({\hat{\theta }}_{CLS}\left(m\right)\right)\overset{d}{\to }\sum_{j=1}^{p+1}\Lambda_{j}\chi^{2}(1)$$

Thus, it is known that $\Lambda_{j}$ is the eigenvalue of the matrix $\Sigma_{11}^{\frac{1}{2}}\Sigma_{*}^{\prime }\Sigma_{11}^{-1}\Sigma_{*}\Sigma_{11}^{\frac{1}{2}}$, and $\sum_{j=1}^{p+1}\Lambda_{j}=trace\left(\Omega \right)=trace\left(\Sigma_{11}^{\frac{1}{2}}\Sigma_{*}^{\prime }\Sigma_{11}^{-1}\Sigma_{*}\Sigma_{11}^{\frac{1}{2}}\right)$. □

**Proof** **of** **Theorem** **2.** Due to assumptions (A1) and (A2), following steps similar to those in Lemma 1 of Zhang, Wang, and Zhu [7], we know that $E\left({\left\|\Psi_{t}\left(\tilde{\theta }\right)\right\|}^{2+\frac{\delta }{2}}\right)$ is bounded above. For any ${\tilde{\theta }}_{1}$ in the neighborhood of ${\tilde{\theta }}^{*}\neq {\tilde{\theta }}_{0}$, by applying the Markov inequality, we have:

$$\sum_{i=1}^{\infty }P\left({\left\|\Psi_{t}\left({\tilde{\theta }}_{1}\right)\right\|}^{2}>i\right)\leq \sum_{i=1}^{\infty }\frac{E\left({\left\|\Psi_{t}\left({\tilde{\theta }}_{1}\right)\right\|}^{2+\frac{\delta }{2}}\right)}{i^{1+\frac{\delta }{4}}}<\infty$$

By applying the Borel–Cantelli lemma, we can always find a sufficiently large natural number *N* such that for any i>N, $\left\|\Psi_{t}\left(\theta_{1}\right)\right\|\leq i^{-\frac{1}{2}}$ holds with probability 1. This further implies that $\underset{1\leq t\leq T}{\max}\left\|\Psi_{t}\left({\tilde{\theta }}_{1}\right)\right\|=o_{p}\left(T^{\frac{1}{2}}\right)$. Therefore:

$$H\left({\tilde{\theta }}_{1}\right)={\left(\sum_{t=p+1}^{T}\Psi_{t}\left({\tilde{\theta }}_{1}\right)\right)}^{\prime }{\left(\sum_{t=p+1}^{T}\Psi_{t}\left({\tilde{\theta }}_{1}\right)\Psi_{t}{\left({\tilde{\theta }}_{1}\right)}^{\prime }\right)}^{-1}\left(\sum_{t=p+1}^{T}\Psi_{t}\left({\tilde{\theta }}_{1}\right)\right)\;\geq {\left(\sum_{t=p+1}^{T}\Psi_{t}\left({\tilde{\theta }}_{1}\right)\right)}^{\prime }{\left(\underset{t}{\max}\left\|\Psi_{t}\left({\tilde{\theta }}_{1}\right)\right\|1^{\prime }\sum_{t=p+1}^{T}\Psi_{t}{\left({\tilde{\theta }}_{1}\right)}^{\prime }\right)}^{-1}\left(\sum_{t=p+1}^{T}\Psi_{t}\left({\tilde{\theta }}_{1}\right)\right)$$

where $1={\left(1,1,\ldots ,1\right)}^{\prime }$. Due to assumption (A3), $\sum_{t=p+1}^{T}\Psi_{t}\left({\tilde{\theta }}_{1}\right)=O_{p}(T)$, we can deduce that:

$$T^{-\frac{1}{2}}H\left({\tilde{\theta }}_{1}\right)\to \infty$$

 □

**Lemma** **A1.**

$H\left(m_{0}\right)=o_{p}(log(T)).$

Given:

$$H\left(m_{0}\right)={\left(\sum_{t=p+1}^{T}\Psi_{t}\left({\hat{\theta }}_{CLS}\left(m_{0}\right)\right)\right)}^{\prime }{\left(\sum_{t=p+1}^{T}\Psi_{t}\left({\hat{\theta }}_{CLS}\left(m_{0}\right)\right)\Psi_{t}{\left({\hat{\theta }}_{CLS}\left(m_{0}\right)\right)}^{\prime }\right)}^{-1}\left(\sum_{t=p+1}^{T}\Psi_{t}\left({\hat{\theta }}_{CLS}\left(m_{0}\right)\right)\right)$$

and

$$\begin{array}{ll} \sum_{t=p+1}^{T}\Psi_{t}\left({\hat{\theta }}_{CLS}\left(m_{0}\right)\right)= & \sum_{t=p+1}^{T}\Psi_{t}\left({\tilde{\theta }}_{0}\right)+{\left(\sum_{t=p+1}^{T}\frac{\partial \Psi_{t}\left({\tilde{\theta }}_{0}\right)}{\partial {\tilde{\theta }}_{\left(1\right)}^{\prime }\left(m\right)}\right)}^{-1}\left({\hat{\theta }}_{\left(1\right),CLS}^{}\left(m\right)-{\tilde{\theta }}_{\left(1\right)}\left(m_{0}\right)\right)+o_{p}\left(1\right)\\ & ≝Q_{t}({\tilde{\theta }}_{0}) \end{array}$$

$$\frac{1}{T}\sum_{t=p+1}^{T}\Psi_{t}\left({\hat{\theta }}_{CLS}\left(m_{0}\right)\right)\Psi_{t}{\left({\hat{\theta }}_{CLS}\left(m_{0}\right)\right)}^{\prime }=\Sigma_{11}+o_{p}(1)$$

We can deduce that:

$$H\left(m_{0}\right)=\frac{1}{T}Q_{t}({\tilde{\theta }}_{0})\Sigma_{11}^{-1}Q_{t}({\tilde{\theta }}_{0})+o_{p}(1)$$

Let $V=Cov\left(\frac{1}{T}\sum_{t=p+1}^{T}\Psi_{t}\left({\tilde{\theta }}_{0}\right)+E\left(\frac{\partial \Psi_{t}\left({\tilde{\theta }}_{0}\right)}{\partial {\tilde{\theta }}_{\left(1\right)}^{\prime }\left(m\right)}\right)\left({\hat{\theta }}_{\left(1\right),CLS}^{}\left(m\right)-{\tilde{\theta }}_{\left(1\right),0}\left(m\right)\right)\right)$ Notice that:

$$P\left(H\left(m_{0}\right)\geq log(T)\right)\leq \frac{E\left(\frac{1}{T}Q_{t}\left({\tilde{\theta }}_{0}\right)\Sigma_{11}^{-1}Q_{t}\left({\tilde{\theta }}_{0}\right)\right)}{log(T)}=\frac{trace\left(\Sigma_{11}^{-1}V\right)}{log(T)}$$

Using Lemma A2 below, we can conclude that $H\left(m_{0}\right)=o_{p}(logT)$.

**Lemma** **A2.** $trace\left(\Sigma_{11}^{-1}V\right)=O_{p}(1)$.

Because both $\Sigma_{11}^{-1}$ and V are semi-positive definite matrices:

$$0\leq trace\left(\Sigma_{11}^{-1}V\right)\leq trace\left(\Sigma_{11}^{-1}\right)trace(V)$$

As $\Sigma_{11}^{-1}=O_{p}(1)$. Note that $\left({\hat{\theta }}_{\left(1\right),CLS}^{}\left(m\right)-{\tilde{\theta }}_{\left(1\right),0}\left(m\right)\right)=O_{p}\left(T^{-\frac{1}{2}}\right)$, and according to Lemma 1, $\frac{1}{T}\sum_{t=p+1}^{T}\Psi_{t}\left({\tilde{\theta }}_{0}\right)=O_{p}\left(1\right)$. Therefore, the proof is complete.

**Proof** **of** **Theorem** **3.** We divide the proof of this theorem into two parts:

(1) If $m_{0}⊈m$, by applying Theorem 1 and Lemma A1 to $m_{0}$, and Theorem 2 to m, we know that for $P_{T}=O\left(T^{\frac{1}{2}}\right)$ and $\frac{log(T)}{P_{T}}\to 0$:
  $$H\left({\hat{\theta }}_{CLS}\left(m\right)\right)+P_{T}\cdot \left|m\right|-H\left({\hat{\theta }}_{CLS}\left(m_{0}\right)\right)-P_{T}\cdot \left|m_{0}\right|\to \infty$$
(2) If $m_{0}\subseteq m$, then by applying Theorem 1 and Lemma A1 to both $m_{0}$ and m, we know that for $P_{T}\to \infty$:
  $$H\left({\hat{\theta }}_{CLS}\left(m\right)\right)+P_{T}\cdot \left|m\right|-H\left({\hat{\theta }}_{CLS}\left(m_{0}\right)\right)-P_{T}\cdot \left|m_{0}\right|\;=P_{T}\left(\left|m\right|-\left|m_{0}\right|\right)+o_{p}(log(T))\to \infty$$
  Therefore,
  $$P\left(\min\left(H\left({\hat{\theta }}_{CLS}\left(m\right)\right)+P_{T}\cdot \left|m\right|:m\neq m_{o}\right)>H\left({\hat{\theta }}_{CLS}\left(m_{0}\right)\right)+P_{T}\cdot \left|m_{0}\right|\right)\to 1$$
   □

**Proof** **of** **Theorem** **4.** Following the steps in Diop and Kengne [21], we have: For $x={\left(x_{i}\right)}_{1\leq i\leq p+1}$, $x_{i}\in R$, define:

$$F_{T}\left(x\right)=P\left(\underset{1\leq i\leq p+1}{⋂}\sqrt{T}{\left({\hat{\theta }}_{CLS}\left(\hat{m}\right)-{\tilde{\theta }}_{0}\right)}_{i}\leq x_{i}\right)$$

Then we have:

$$F_{T}\left(x\right)=P\left(\underset{1\leq i\leq p+1}{⋂}\sqrt{T}{\left({\hat{\theta }}_{CLS}\left(\hat{m}\right)-{\tilde{\theta }}_{0}\right)}_{i}\leq x_{i}|\hat{m}=m_{o}\right)P\left(\hat{m}=m_{o}\right)\;\;\;\;\;+P\left(\underset{1\leq i\leq p+1}{⋂}\sqrt{T}{\left({\hat{\theta }}_{CLS}\left(\hat{m}\right)-{\tilde{\theta }}_{0}\right)}_{i}\leq x_{i}|\hat{m}\neq m_{o}\right)P\left(\hat{m}\neq m_{o}\right)$$

According to Theorem 3, as T→∞:

$$P\left(\hat{m}=m_{0}\right)\to 1,\;P\left(\hat{m}\neq m_{0}\right)\to 0$$

Therefore:

$$P\left(\underset{1\leq i\leq p+1}{⋂}\sqrt{T}{\left({\hat{\theta }}_{CLS}\left(\hat{m}\right)-{\tilde{\theta }}_{0}\right)}_{i}\leq x_{i}|\hat{m}\neq m_{o}\right)P\left(\hat{m}\neq m_{o}\right)\to 0$$

Hence:

$$F_{T}\left(x\right)=P\left(\left\{\underset{i\in m_{o}}{⋂}\sqrt{T}{\left({\hat{\theta }}_{CLS}\left(\hat{m}\right)-{\tilde{\theta }}_{0}\right)}_{i}\leq x_{i}\right\}⋂\left\{\underset{i\notin m_{o}}{⋂}\sqrt{T}{\left({\hat{\theta }}_{CLS}\left(\hat{m}\right)-{\tilde{\theta }}_{0}\right)}_{i}\leq x_{i}\right\}\right)$$

Since $\tilde{\theta }\left(m_{0}\right)\in \tilde{\Theta }(m_{0})$, ${\left({\left({\hat{\theta }}_{CLS}\left(m_{0}\right)\right)}_{i}\right)}_{i\notin m_{0}}={\left({\tilde{\theta }}_{i}\right)}_{i\notin m_{0}}=0$ and ${\left(x_{i}\right)}_{i\notin m_{0}}$ is a set of real numbers, i.e., ${\left(x_{i}\right)}_{i\notin m_{0}}<\infty$, then by Lemma 1:

$$P\left(\left\{\underset{i\in m_{o}}{⋂}\sqrt{T}{\left({\hat{\theta }}_{CLS}\left(\hat{m}\right)-{\tilde{\theta }}_{0}\right)}_{i}\leq x_{i}\right\}⋂\left\{\underset{i\notin m_{o}}{⋂}\sqrt{T}{\left({\hat{\theta }}_{CLS}\left(\hat{m}\right)-{\tilde{\theta }}_{0}\right)}_{i}\leq x_{i}\right\}\right)\;=P\left(\underset{i\in m_{o}}{⋂}\sqrt{T}{\left({\hat{\theta }}_{CLS}\left(\hat{m}\right)-{\tilde{\theta }}_{0}\right)}_{i}\leq x_{i}\right)+o_{p}(1)\;\to P\left({\left(\Sigma \left({\tilde{\theta }}_{0}\right)\right)}^{-\frac{1}{2}}Z\leq {\left(x_{i}\right)}_{i\in m_{0}}\right)$$

where $\Sigma \left({\tilde{\theta }}_{0}\right)=V^{-1}({\tilde{\theta }}_{0})W({\tilde{\theta }}_{0})V^{-1}({\tilde{\theta }}_{0})$, and Z is a standard normal random vector of dimension $\left|m_{0}\right|$ □

### Appendix A.2. Complementary Tables

**Table A1:** Frequency of model selection for INAR model of order 1 by the penalized criterion (6).

$Y_{t}=\phi_{2}^{\left(t\right)}\circ Y_{t-2}+Z_{t}$										
$\phi_{2}=0.3$			Models to Be Selected							
T	Coef	$P_{T}$	i.i.d.	$y_{t-1}$	$y_{t-2}$	$y_{t-3}$	$y_{t-1}{,y}_{t-2}$	$y_{t-1}{,y}_{t-3}$	$y_{t-2}{,y}_{t-3}$	$y_{t-1}{,y}_{t-2}{,y}_{t-3}$
100	Fixed	$T^{1/3}$	0.283	0.025	**0.647**	0.006	0.016	0.001	0.022	0
		log(T)	0.281	0.025	**0.648**	0.006	0.016	0.001	0.023	0
		$T^{1/5}$	0.08	0.025	**0.691**	0.015	0.088	0.008	0.085	0.008
	Uniform	$T^{1/3}$	0.304	0.033	**0.618**	0.01	0.011	0	0.023	0.001
		log(T)	0.297	0.034	**0.623**	0.011	0.011	0	0.023	0.001
		$T^{1/5}$	0.104	0.038	**0.652**	0.015	0.087	0.003	0.085	0.016
	Beta	$T^{1/3}$	0.287	0.029	**0.64**	0.011	0.011	0.001	0.021	0
		log(T)	0.281	0.029	**0.645**	0.011	0.011	0.001	0.022	0
		$T^{1/5}$	0.118	0.032	**0.666**	0.01	0.076	0.005	0.081	0.012
200	Fixed	$T^{1/3}$	0.058	0.004	**0.902**	0.001	0.017	0	0.018	0
		log(T)	0.045	0.003	**0.902**	0.001	0.021	0	0.024	0.001
		$T^{1/5}$	0.007	0	**0.804**	0.003	0.081	0	0.092	0.013
	Uniform	$T^{1/3}$	0.091	0.006	**0.882**	0.002	0.006	0	0.013	0
		log(T)	0.071	0.007	**0.894**	0.002	0.009	0	0.013	0
		$T^{1/5}$	0.017	0.002	**0.809**	0	0.074	0	0.09	0.008
	Beta	$T^{1/3}$	0.059	0.004	**0.912**	0.001	0.011	0	0.012	0.001
		log(T)	0.044	0.003	**0.911**	0.001	0.018	0	0.021	0.002
		$T^{1/5}$	0.009	0.001	**0.803**	0.001	0.081	0	0.092	0.013
300	Fixed	$T^{1/3}$	0.013	0	**0.97**	0.001	0.008	0	0.008	0
		log(T)	0.007	0	**0.96**	0.001	0.015	0	0.017	0
		$T^{1/5}$	0.001	0	**0.836**	0	0.076	0	0.077	0.01
	Uniform	$T^{1/3}$	0.017	0	**0.956**	0	0.016	0	0.01	0.001
		log(T)	0.009	0	**0.94**	0	0.025	0	0.024	0.002
		$T^{1/5}$	0	0	**0.839**	0	0.081	0	0.264	0.016
	Beta	$T^{1/3}$	0.011	0	**0.967**	0	0.012	0	0.01	0
		log(T)	0.008	0	**0.956**	0	0.019	0	0.017	0
		$T^{1/5}$	0	0	**0.857**	0	0.078	0	0.058	0.007
500	Fixed	$T^{1/3}$	0	0	**0.99**	0	0.008	0	0.002	0
		log(T)	0	0	**0.977**	0	0.012	0	0.011	0
		$T^{1/5}$	0	0	**0.868**	0	0.054	0	0.066	0.012
	Uniform	$T^{1/3}$	0	0	**0.991**	0	0.003	0	0.006	0
		log(T)	0	0	**0.974**	0	0.01	0	0.016	0
		$T^{1/5}$	0	0	**0.874**	0	0.047	0	0.073	0.006
	Beta	$T^{1/3}$	0	0	**0.993**	0	0.004	0	0.003	0
		log(T)	0	0	**0.976**	0	0.011	0	0.013	0
		$T^{1/5}$	0	0	**0.879**	0	0.056	0	0.058	0.007
1000	Fixed	$T^{1/3}$	0	0	**0.998**	0	0	0	0.002	0
		log(T)	0	0	**0.987**	0	0.006	0	0.007	0
		$T^{1/5}$	0	0	**0.916**	0	0.04	0	0.043	0
	Uniform	$T^{1/3}$	0	0	**0.996**	0	0.001	0	0.003	0
		log(T)	0	0	**0.978**	0	0.012	0	0.01	0
		$T^{1/5}$	0	0	**0.914**	0	0.045	0	0.036	0.005
	Beta	$T^{1/3}$	0	0	**0.998**	0	0.001	0	0.001	0
		log(T)	0	0	**0.987**	0	0.005	0	0.008	0
		$T^{1/5}$	0	0	**0.901**	0	0.041	0	0.055	0.003

**Table A2:** Frequency of model selection for INARCH model of order 1 by the penalized criterion (6).

$Y_{t}|F_{t-1}\sim Poisson(\lambda_{t})$										
$\lambda_{t}=\phi_{0}+\phi_{1}Y_{t-1}$										
$\phi_{1}=0.3$			Models to Be Selected							
T	Criterion	$P_{T}$	i.i.d.	$y_{t-1}$	$y_{t-2}$	$y_{t-3}$	$y_{t-1}{,y}_{t-2}$	$y_{t-1}{,y}_{t-3}$	$y_{t-2}{,y}_{t-3}$	$y_{t-1}{,y}_{t-2}{,y}_{t-3}$
100	$H+P_{T}\cdot \left|m\right|$	$T^{1/3}$	0.09	**0.849**	0.003	0.02	0.032	0.022	0	0.002
		log(T)	0.087	**0.85**	0.003	0.002	0.033	0.023	0	0.002
		$T^{1/5}$	0.024	**0.746**	0.006	0.002	0.109	0.094	0	0.019
	AIC		0.011	**0.713**	0.005	0.001	0.125	0.116	0	0.029
	BIC		0.06	**0.888**	0.002	0.003	0.023	0.023	0	0.001
200	$H+P_{T}\cdot \left|m\right|$	$T^{1/3}$	0.001	**0.958**	0	0	0.017	0.023	0	0.001
		log(T)	0.001	**0.947**	0	0	0.022	0.029	0	0.001
		$T^{1/5}$	0	**0.821**	0	0	0.071	0.09	0	0.018
	AIC		0	**0.719**	0	0	0.113	0.129	0	0.039
	BIC		0	**0.959**	0	0	0.019	0.021	0	0.001
300	$H+P_{T}\cdot \left|m\right|$	$T^{1/3}$	0	**0.986**	0	0	0.005	0.009	0	0
		log(T)	0	**0.978**	0	0	0.01	0.012	0	0
		$T^{1/5}$	0	**0.854**	0	0	0.065	0.075	0	0
	AIC		0	**0.707**	0	0	0.124	0.148	0	0.021
	BIC		0	**0.977**	0	0	0.009	0.014	0	0
500	$H+P_{T}\cdot \left|m\right|$	$T^{1/3}$	0	**0.994**	0	0	0.004	0.002	0	0
		log(T)	0	**0.978**	0	0	0.012	0.01	0	0
		$T^{1/5}$	0	**0.878**	0	0	0.06	0.057	0	0.005
	AIC		0	**0.704**	0	0	0.135	0.126	0	0.035
	BIC		0	**0.973**	0	0	0.017	0.01	0	0
1000	$H+P_{T}\cdot \left|m\right|$	$T^{1/3}$	0	**0.997**	0	0	0.02	0.01	0	0
		log(T)	0	**0.979**	0	0	0.008	0.013	0	0
		$T^{1/5}$	0	**0.904**	0	0	0.042	0.05	0	0.004
	AIC		0	**0.701**	0	0	0.13	0.142	0	0.027
	BIC		0	**0.976**	0	0	0.008	0.016	0	0

**Table A3:** Frequency of model selection for AR model by the penalized criterion (6) with $Z_{t}$ misspecification.

$Y_{t}=\phi_{0}+\phi_{1}Y_{t-1}+\phi_{3}Y_{t-3}+Z_{t}$										
$Z_{t}\sim Uniform([-2,2])$										
$\phi_{1}=0.4$ $,\;\phi_{3}=0.2$			Models to Be Selected							
T	Criterion	$P_{T}$	i.i.d.	$y_{t-1}$	$y_{t-2}$	$y_{t-3}$	$y_{t-1}{,y}_{t-2}$	$y_{t-1}{,y}_{t-3}$	$y_{t-2}{,y}_{t-3}$	$y_{t-1}{,y}_{t-2}{,y}_{t-3}$
100	$H+P_{T}\cdot \left|m\right|$	$T^{1/3}$	0.046	0.595	0.001	0.016	0.016	**0.317**	0.001	0.008
		log(T)	0.046	0.59	0.001	0.015	0.017	**0.322**	0.001	0.008
		$T^{1/5}$	0.003	0.366	0.001	0.008	0.039	**0.516**	0	0.067
	AIC		0.003	0.269	0.001	0.003	0.059	**0.554**	0	0.111
	BIC		0.026	0.578	0.001	0.012	0.021	**0.349**	0.001	0.012
200	$H+P_{T}\cdot \left|m\right|$	$T^{1/3}$	0	0.356	0	0	0.008	**0.63**	0	0.006
		log(T)	0	0.308	0	0	0.012	**0.669**	0	0.011
		$T^{1/5}$	0	0.115	0	0	0.026	**0.794**	0	0.065
	AIC		0	0.069	0	0	0.027	**0.787**	0	0.117
	BIC		0	0.283	0	0	0.014	**0.691**	0	0.012
300	$H+P_{T}\cdot \left|m\right|$	$T^{1/3}$	0	0.157	0	0	0.002	**0.832**	0	0.009
		log(T)	0	0.105	0	0	0.005	**0.875**	0	0.015
		$T^{1/5}$	0	0.029	0	0	0.009	**0.901**	0	0.061
	AIC		0	0.01	0	0	0.008	**0.851**	0	0.131
	BIC		0	0.098	0	0	0.006	**0.883**	0	0.013
500	$H+P_{T}\cdot \left|m\right|$	$T^{1/3}$	0	0.029	0	0	0.01	**0.966**	0	0.004
		log(T)	0	0.013	0	0	0.01	**0.975**	0	0.011
		$T^{1/5}$	0	0.002	0	0	0.01	**0.933**	0	0.064
	AIC		0	0	0	0	0.01	**0.841**	0	0.158
	BIC		0	0.011	0	0	0.02	**0.977**	0	0.01
1000	$H+P_{T}\cdot \left|m\right|$	$T^{1/3}$	0	0	0	0	0	**0.999**	0	0.001
		log(T)	0	0	0	0	0	**0.992**	0	0.008
		$T^{1/5}$	0	0	0	0	0	**0.962**	0	0.038
	AIC		0	0	0	0	0	**0.864**	0	0.136
	BIC		0	0	0	0	0	**0.992**	0	0.008
